# (−)- and (+)-Securidanes A and B, Natural Triarylmethane Enantiomers: Structure and Bioinspired Total Synthesis

**DOI:** 10.1155/2018/2674182

**Published:** 2018-09-05

**Authors:** B. Zhou, D. X. Liu, X. J. Yuan, J. Y. Li, Y. C. Xu, J. Li, Y. Li, J. M. Yue

**Affiliations:** ^1^State Key Laboratory of Drug Research, Shanghai Institute of Materia Medica, Chinese Academy of Sciences, 555 Zuchongzhi Road, Shanghai 201203, China; ^2^State Key Laboratory of Applied Organic Chemistry, College of Chemistry and Chemical Engineering, Lanzhou University, Lanzhou 730000, China

## Abstract

Two pairs of enantiomers, (−) and (+)-securidanes A (**1** and** 2**) and B (**3** and** 4**) featuring unprecedented triarylmethane (TAM) skeletons, were isolated from* Securidaca inappendiculata*. Their structures were established by spectroscopic data, X-ray crystallography, and CD analysis. A plausible biosynthetic pathway for** 1**−**4** based on the co-isolated precursors was proposed. Bioinspired total synthesis of** 1**−**4** was completed in high yield, which in turn corroborated the biosynthetic hypothesis. Compounds** 1**−**4** showed good inhibition against protein tyrosine phosphatase 1B (PTP1B). The molecular docking demonstrated that the strongest inhibitor** 3** (IC_50_ = 7.52* μ*M) reaches deeper into the binding pocket and has an additional H-bond.

## 1. Introduction

Compounds incorporating the triarylmethane (TAM) motif are well recognized in materials science, such as fluorescent probes, organic dyes, and metal ion sensors [1–5]. It is particularly interesting that TAM derivatives have also demonstrated a broad spectrum of biological significance including anticancer, K^+^ channel blocking, histidine protein kinase inhibitory, and antiparasitic, antiviral, and antitubercular activities [6–10]. Given the important roles of TAMs in materials science and medicinal chemistry, this compound class has attracted widespread attention in the area of organic chemistry and a number of synthetic methods have been developed by involving the key steps of Friedel−Crafts reaction, transition-metal-catalytic cross-coupling reaction, and reductive dehydroxylation of triarymethanol derivatives, including a few approaches of asymmetric catalysis [11–25], which has led to the synthesis of a large array of TAMs. However, only a very limited number of flavonoid derivatives with a TAM motif have been identified from natural products hitherto [26–29].

**Figure 1:**
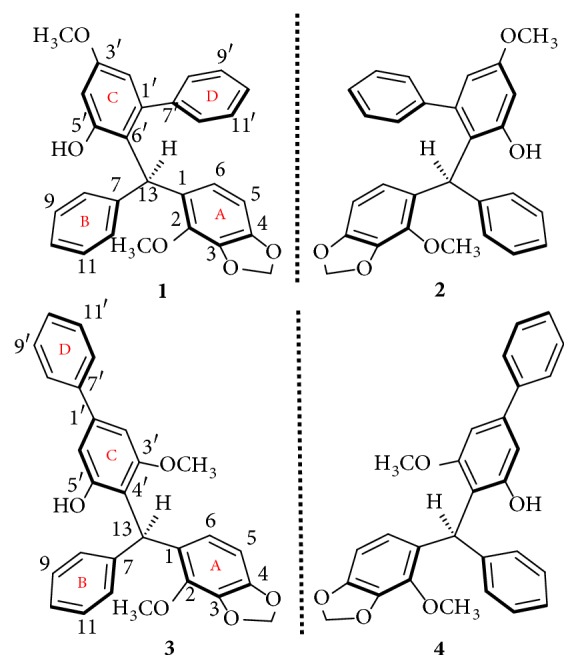
Structures of compounds** 1**–**4**.

The plant* Securidaca inappendiculata *Hassk. (Polygalaceae) is mainly distributed in the south of China and the tropical regions of Asia. The whole plants have been applied in the remedies of traditional Chinese medicine [30], from which a number of xanthone, benzophenone, and sterol derivatives were identified with anti-inflammatory, anti-HIV, and MAO inhibitory activities [31–34]. In this study, two enantiomeric pairs, (−)- and (+)-securidanes A (**1** and** 2**) and B (**3** and** 4**) featuring new TAM skeletons, were obtained as optically pure compounds by chiral separation from the stems of* S*.* inappendiculata* (Figure 1). Compounds** 1**−**4** biosynthetically descended from a diphenylmethane and a diphenyl derivative are unprecedented, and a plausible biosynthetic pathway based on the coisolated precursor** 5** was proposed. Bioinspired total synthesis of** 1**−**4** was completed in high yield, which in turn corroborated the biosynthetic hypothesis. PTP1B plays a profound role in cell regulation, growth, and the onset of human diseases. Its overexpression causes persistent dephosphorylation of insulin receptor, stimulating the insulin-resistant phenotype in type 2 diabetes and obesity [35]. PTP1B has thus been considered as a potential therapeutic target for type 2 diabetes and obesity [36, 37]. Compounds** 1**−**4** showed PTP1B inhibitions with IC_50_ values ranging from 7.5 to 15.6* μ*m. The molecular docking showed that the strongest inhibitor** 3** reaches deeper into the binding pocket and has an additional H-bond. Herein, we present the isolation, chiral separation, structural elucidation, biological evaluation, and bioinspired total synthesis of** 1**–**4**.

## 2. Results

(−)-Securidane A (**1**) was obtained as colorless crystals (in MeOH) with [*α*]^22^_D_  −76.9 (*c* 0.42, MeOH). The molecular formula, C_28_H_24_O_5_ with 17 double-bond equivalents (DBEs), was determined by the HRESIMS ion at* m/z* 441.1700 [M + H]^+^ (calcd 441.1697) and the NMR data. Its IR absorption bands showed the presence of hydroxy (3487 cm^−1^) and aromatic (1613 and 1576 cm^−1^) functionalities. The ^1^H NMR data ([Sec supplementary-material-1]) displayed the diagnostic resonances of two methoxy and one methylenedioxy groups. The ^13^C NMR data ([Sec supplementary-material-1]) with the aid of DEPT experiments revealed the existence of two methyls, one methylene, 15 methines (14 sp^2^ and one sp^3^), and 10 sp^2^ quaternary carbons. Comprehensive analysis of ^1^H and ^13^C NMR data indicated the presence of four phenyl groups (two mono- and two tetrasubstituted), which accounted for 16 out of the 17 DBEs, and the remaining one DBE required one more ring in the molecule. A singlet proton signal at *δ*_H_ 5.40 that did not show correlations with any carbons in the HSQC spectrum ([Sec supplementary-material-1]) was assigned to a hydroxy group. Particularly, the diagnostic sp^3^ methane group (*δ*_H_ 5.58, s; *δ*_C_ 45.0) suggested that it is a TAM derivative.

**Figure 2:**
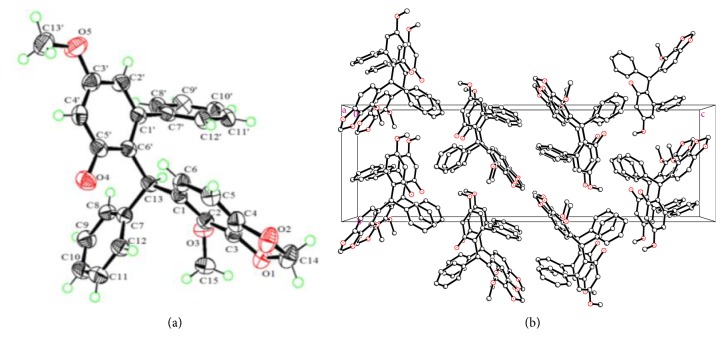
(a) X-ray structure of** 1**. (b) Molecule assembly in crystals.

**Figure 3:**
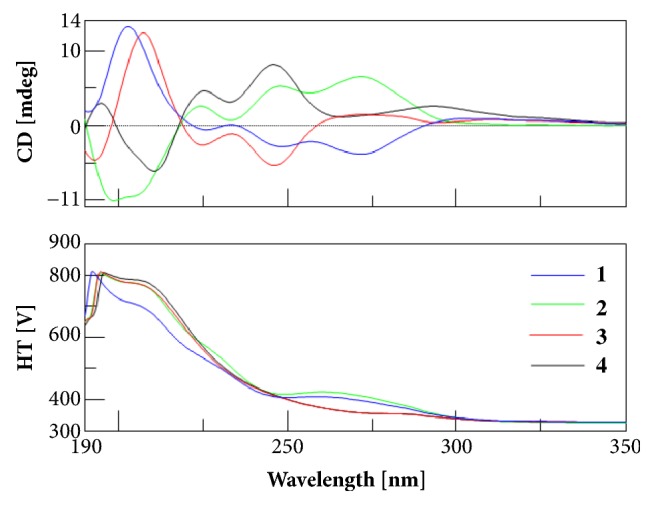
CD spectra of** 1**–**4**.

**Figure 4:**
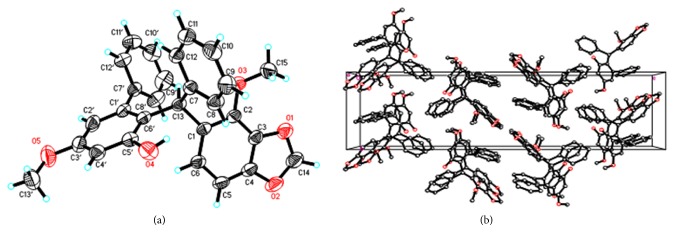
(a) X-ray structure of** 2**. (b) Molecule assembly in crystals.

The planar structure of** 1** was established by 2D NMR analysis ([Sec supplementary-material-1]). Three proton-bearing coupling fragments as drawn in bold bonds were revealed by ^1^H-^1^H COSY spectrum. In the HMBC spectrum, the key correlations from H-13 to C-1, C-7, and C-6′ attached three phenyls to C-13. The HMBC correlations of H-12′/C-1′ and H-2′/C-7′ placed the remaining phenyl unit at C-1′ to form a diphenyl motif via the C-1′−C-7′ bond. Two methoxyls were located at C-2 and C-3′ by the HMBCs of 2-OCH_3_/C-2 and 3′-OCH_3_/C-3′, respectively. The only hydroxy group was assigned to C-5′ by the key HMBC correlation of 5′-OH/C-5′. The downfield shifted methylene signals (*δ*_H_ 5.93, 5.95; *δ*_C_ 101.2) was assigned to a 3,4-methylenedioxy group by the chemical shifts and the HMBCs from the two protons to both C-3 and C-4. The planar structure of** 1** was thus delineated. The absolute configuration of** 1 **was unambiguously determined as 13*R* by a single crystal X-ray diffraction study (Figure 2), in which the anomalous dispersion of Cu K*α* radiation was applied and the absolute structure parameter of −0.13(7) was acquired.

(+)-Securidane A (**2**) shared the same molecular formula and identical NMR data with** 1** ([Sec supplementary-material-1], Figures [Sec supplementary-material-1] and [Sec supplementary-material-1]), but had an opposite specific rotation [*α*]^22^_D_ +72.3 (*c* 0.48, MeOH) and CD curve to that of** 1** (Figure 3), indicating that it is the enantiomer of** 1** and 13*S*-configured. Crystallization of** 2** from methanol allowed for a successful performance of X-ray diffraction analysis (Figure 4), which not only confirmed its absolute configuration [absolute structure parameter 0.04(10)], but also provided solid evidence to understand the conformation and molecular assembling patterns of enantiomeric pair of triarylmethane-type compounds** 1** and** 2** in the solid state.

(−)-Securidane B (**3**), [*α*]^22^_D_  −43.5 (*c* 0.37, MeOH), had a molecular formula of C_28_H_24_O_5_ as determined by the HRESIMS ion at* m/z *441.1707 [M + H]^+^ (calcd 441.1697). The NMR data ([Sec supplementary-material-1]) showing great similarities to those of** 1** suggested that it is also a TAM analogue. The major differences observed in its ^13^C NMR spectrum ([Sec supplementary-material-1]) were the C-4′ and C-6′. The quaternary C-4′ (*δ*_C_ 116.7) and the methine C-6′(*δ*_C_ 102.4) resonated downfield (Δ*δ*_C_ +14.4) and upfield (Δ*δ*_C_  −17.0), respectively, as compared to those of** 1**, indicative of an alternative conjugation between the diphenyl and diphenylmethane motifs. The planar structure of** 3** was finally constructed by 2D NMR spectra (Figures [Sec supplementary-material-1] and [Sec supplementary-material-1]–[Sec supplementary-material-1]), especially HMBC data. The linkage between two motifs via a C-13−C-4′ bond was confirmed by the key HMBC correlation from H-13 (*δ*_H_ 6.30, s) to C-4′. The substituted patterns in two motifs were assigned to be identical to those of** 1** by the HMBC correlations. The tendency of its CD curve (Figure 3) is compatible to that of** 1**, suggesting that** 3** had a 13*R-*configuration. This was supported by its negative specific rotation as compared to** 1**.

(+)-Securidane B (**4)** possessed the same molecular formula C_28_H_24_O_5_ and the identical NMR data as those of** 3** ([Sec supplementary-material-1], Figures [Sec supplementary-material-1] and [Sec supplementary-material-1]), while showing opposite CD curve (Figure 3) and specific rotation of [*α*]^22^_D_ = +42.3 to those of** 3**. It was thus assigned as the enantiomer of** 3 **to be 13*S*-configured.

A direct LC-ESIMS analysis of the fresh ethanolic extract of the plant stems showed the presence of the diagnostic ion peaks at* m/z* 441 [M + H]^+^ and 439 [M − H]^−^ for** 1**−**4** ([Sec supplementary-material-1]), indicating that they are not artifacts produced in the separation.

**Scheme 1:**
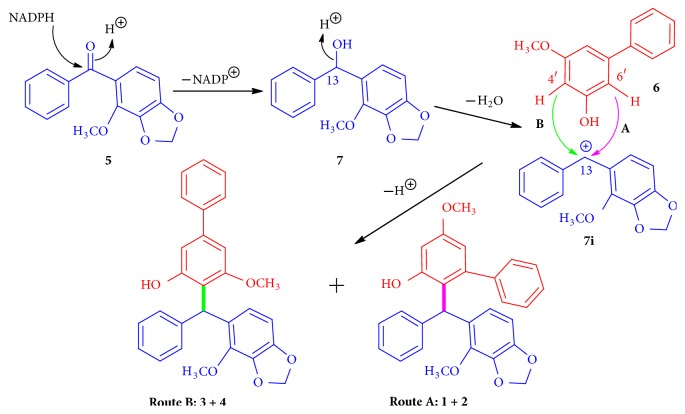
Plausible biosynthetic pathway of compounds** 1**–**4**.

A possible biosynthetic pathway for** 1**−**4 **was proposed (Scheme 1). The co-isolate** 5** [9] and a natural product** 6** [38] were served as the biosynthetic precursors. Although** 6** has not been isolated in this study, it is presumed to exist in the plant either in a low concentration or with a very short lifespan after production. Reduction of** 5** by NADPH would produce the key intermediate** 7**, which was readily transformed to a very stable carbocation** 7i**. Nucleophilic attack of C-4′ or C-6′ of** 6** on to** 7i** via electrophilic aromatic substitution reaction would produce (−)- and (+)-securidanes A (**1** and** 2**) (route A in pink) and B (**3** and** 4**) (route B in green).

**Scheme 2:**
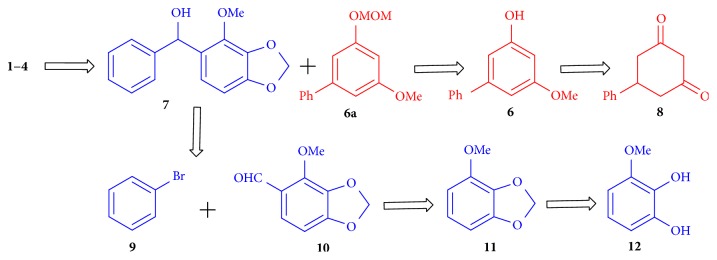
Bioinspired retrosynthetic analysis of** 1**−**4**.

To confirm the biosynthetic hypothesis, we carried out a bioinspired total synthesis of** 1**−**4**. The retrosynthetic analysis (Scheme 2) involves a biomimetic assembling of** 6a** and** 7 **via Kim's protocol [25] as the key step to furnish the targeted TAM frameworks of** 1**−**4**, in which** 7** is the key biosynthetic intermediate, and** 6a** is the MOM ether of the other biosynthetic precursor** 6**. Synthesis of fragment** 7** in turn was envisioned to arise from aldehyde** 10** by a Grignard reaction. While the aldehyde** 10** could be made by the treatment of** 11** under formylation condition, biaryl compound** 6a** could be readily prepared from** 8** in two steps.

### 2.1. Synthesis of** 7**

**Scheme 3:**
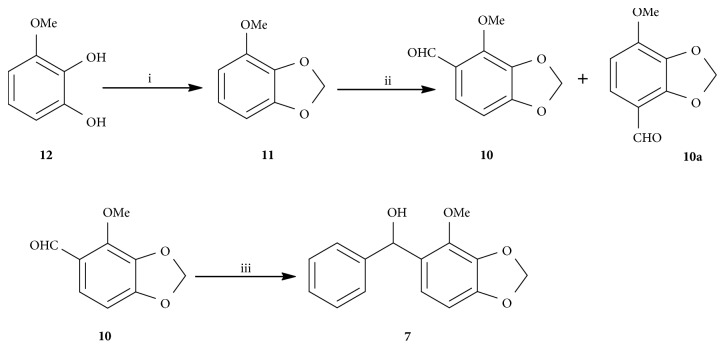
Synthesis of** 7**, (i) NaH, HMPA, CH_2_I_2_, rt, 87%; (ii) DMF, POCl_3_, 100°C, 7 h, 52%; (iii) Mg, I_2_, bromobenzene, THF, rt, 99%.

Compound** 11** was prepared in 87% yield by alkylation of** 12** [39]. Formylation of** 11 **then produced two isomeric aldehydes** 10** and** 10a **in a ratio of 2:1 [40, 41]. Addition of Grignard reagents formed from bromobenzene (**9**) to the aldehyde** 10 **afforded the desired alcohol** 7** in a good yield of 99% (Scheme 3) [42].

### 2.2. Synthesis of** 6a**

**Scheme 4:**
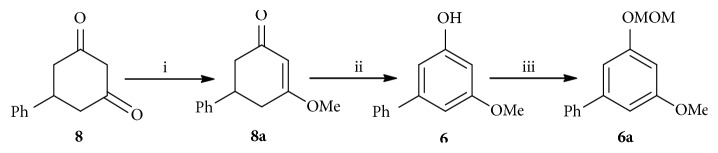
Synthesis of** 6a**, (i) H_2_SO_4_, MeOH, rt, 92%; (ii) AcOH, Hg(OAc)_2_, reflux, 7 h, 60%; (iii) CH_3_OCH_2_Cl, NaH, THF, 0°C to rt, 98%.

Biaryl** 6a** was synthesized from the known starting material** 8 **(Scheme 4). Enolization of** 8** under acidic condition in methanol at room temperature afforded** 8a**, which was then converted into** 6** by refluxing with Hg(OAc)_2_ in AcOH for 7 h [43].** 6a** was finally obtained by protection of the hydroxyl of** 6** with MOM ether [44].

### 2.3. Synthesis of** 1**−**4**

**Scheme 5:**
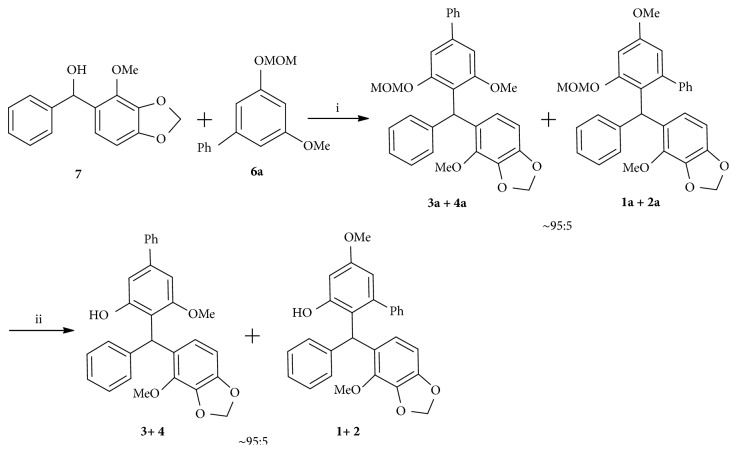
Synthesis of** 1**−**4**, (i) Fe (ClO_4_)_3_•xH_2_O, CH_3_CN, rt, 57%; (ii) HCl, MeOH, reflux, 79%.

With the key fragments** 6a** and** 7** in hand, we next focused on their assembling in the presence of Fe (III) [24] (Scheme 5). The desired coupling products were obtained as a mixture of two racemic pairs** 1a**−**4a**, which were dominated by the racemates** 3a** and** 4a** (ca. 95%, 1:1) with the minor racemic products** 1a** and** 2a** (ca. 5%, 1:1) (determined by HPLC analysis). Deprotection of the MOM ethers [45] afforded a mixture of two pair racemates in 79% total yield, which was further separated into four optically pure compounds** 1**−**4 **by chiral HPLC preparation. The products of chemical synthesis were dominated by (−)- and (+)-securidane B (**3** and** 4**, 95%) due largely to the different steric hindrance in two coupling models, while the natural isolates** 1**−**4** from the plant were accounted for approximately 25% each (Figures [Sec supplementary-material-1]–[Sec supplementary-material-1]). It is suggested that the transition state in the key step of chemical coupling is different from that of the biosynthesis in the plant, and the MOM ether protection group of chemical synthesis is likely an influencing factor for the ratios of the products.

### 2.4. PTP1B Inhibitory Evaluation

Compounds** 1**−**4** were tested for the inhibitory effects on PTP1B enzyme by using an* in vitro* assay [46], and a well-recognized natural PTP1B inhibitor oleanolic acid was used as the positive control (IC_50_ = 4.14 ± 0.59* μ*M). Compounds** 1**−**4** showed remarkable inhibition with IC_50_ values of 15.6 ± 1.37, 12.6 ± 3.68, 7.5 ± 0.74, and 10.5 ± 2.86* μ*M, respectively. This is the first report of TAMs as PTP1B inhibitors.

### 2.5. Molecular Docking

**Figure 5:**
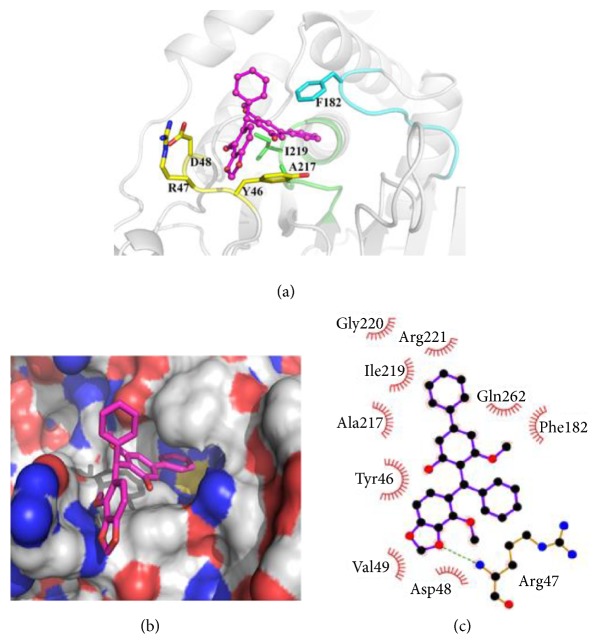
**Docking pose of 3 showing key interactions between the compound and PTP1B. **(**a**) The protein is shown as light grey cartoon, while the catalytic loop, the YRD motif, and the WPD loop are colored* green, yellow*, and* cyan*, respectively;** 3** is represented as a ball-and-stick model with carbon and oxygen colored* magenta* and* red*, respectively. (**b**) The protein is shown by molecular surface and** 3** is represented as sticks. (**c**) Key interactions between** 3** and PTP1B are analyzed by Ligplot+.

The main structural modules furnishing the ligand binding pocket of PTP1B have been demonstrated as the catalytic loop, the YRD motif, and the WPD loop [47]. The molecular docking results (Figures 5, S7, and S8) revealed that the best scoring docking conformations of compounds** 1**−**4** at the binding pocket of PTP1B and their interacting patterns are similar. Taking compound** 3** as an example (Figure 5), ring A participates in hydrophobic interactions with Arg47, Asp48, and Val49 at the YRD motif and forms a hydrogen bond with the main-chain of Arg47. Rings C and D bury into a hydrophobic pocket mostly contributed by Try46 at the YRD motif, Phe182 at the WPD loop, and residues at the catalytic site including Ala217, Ile219, and Gly220. Four compounds interact with the same binding pocket, while** 3** and** 4**, a pair of enantiomers, reach deeper into this hydrophobic pocket as compared to** 1** and** 2.** This may contribute to the higher potency of** 3** and** 4** to PTP1B. The additional hydrogen bond between ring A of** 3** and PTP1B, which is devoid in the complex of** 4** and PTP1B, makes** 3** to be the most potent inhibitor among those four compounds.

## 3. Discussion

In conclusion, we have identified optically pure (−)- and (+)-securidanes A and B (**1**−**4**) featuring unprecedented TAM skeletons from a Chinese medicinal plant* S. inappendiculata*. A plausible biosynthetic pathway for** 1**−**4 **based on the coisolated precursor** 5** was proposed. Bioinspired total synthesis of** 1**−**4** was achieved in high yield, which in turn corroborated the biosynthetic hypothesis. Compounds** 1**−**4** showed good inhibition against PTP1B. Our efforts provide for the first time the chemophysical data for the optically pure TAM analogues, a bioinspired total synthesis of TAMs, and TAMs as PTP1B inhibitors. This finding is of great importance for understanding the biosynthesis of natural TAMs and exploration of their medicinal potency.

## 4. Materials and Methods

### 4.1. General Experimental Procedures

The general experiments were completed according to the reported general procedures with minor modification (Experimental Section, Supporting Information) [48].

#### 4.1.1. Plant Material

The detail information of the plant of* S*.* inappendiculata *was included in the Experimental Section, Supporting Information.

#### 4.1.2. PTP1B Inhibition Assay

A colorimetric assay for the measurement of PTP1B inhibition was performed according to the reported protocols (Experimental Section, Supporting Information) [46, 49].

#### 4.1.3. Molecular Docking

The crystal structure of PTP1B in complex with one of benzotriazole inhibitors (PDB ID: 1Q6P) was used to prepare the receptor structure, and the centroid of the inhibitor was selected as the center of grid boxes [50]. Water molecules, ions, and the inhibitor were deleted before docking performance. The receptor was then prepared using Protein Preparation and Grid Preparation tools in the Schrödinger Maestro interface. As for ligands, the 3D structures of compounds** 1**–**4** were optimized with B3LYP/6-31G^*∗*^ using GAUSSIAN 09 [51, 52]. Molecular docking were performed using the Glide extraprecision mode with default settings [53, 54]. The OPLS-2005 force field was used for minimization and grid generation, while OPLS-2001 was used for docking. Key interactions between compound** 3** and PTP1B were analyzed by Ligplot+ [55].

## Data Availability

All data are available in the manuscript or supplementary materials.

## Supplementary Materials

Supplementary Materials Table S1. Figures S1~S43: general experimental procedures, X-ray crystal data of** 1** and** 2**, biomimetic total synthesis of** 1**–**4**, PTP1B inhibitory assay, and molecular docking of** 1**–**4**.
