# Supplementary material for: (−)- and (+)-Securidanes A and B, Natural Triarylmethane Enantiomers: Structure and Bioinspired Total Synthesis
Source: Research (Wash D C). 2018 Sep 5;2018:2674182. doi: 10.1155/2018/2674182 (PMC6750104; doi:10.1155/2018/2674182)

Supporting Information

(−) and (+)-Securidanes A and B, Natural Triarylmethane Enantiomers: Structure and Bioinspired Total Synthesis

Bin Zhou,[†,§] Dong-Xue Liu,[‡,§] Xiao-Jing Yuan,[†] Jing-Ya Li,[†] Ye-Chun Xu,[†] Jia Li,[†] Ying Li[‡] and Jian-Min Yue*[,†]

[†]State Key Laboratory of Drug Research, Shanghai Institute of Materia Medica, Chinese Academy of Sciences, 555 Zuchongzhi Road, Shanghai 201203, People's Republic of China

[‡] State Key Laboratory of Applied Organic Chemistry, College of Chemistry and Chemical Engineering, Lanzhou University, Lanzhou 730000, People's Republic of China

[§]These authors contributed equally to this work.

* E-mail: jmyue@simm.ac.cn

Table of Contents

Table S1. ^{1}H (500 MHz) and ^{13}C NMR (125 MHz) Data of **1–4** in $CDCl_3$................ 5

Figure S1. ^{1}H–H COSY (bold bond) and key HMBC (red arrows) correlations of **1**.. 6

Figure S2.^{1}H–H COSY (bold bond) and key HMBC (red arrows) correlation of **3**.... 7

Figure S3. HPLC separation of the natural enantiomeric pairs (**1** + **2**) and (**3** + **4**)..... 8

Figure S4. Chiral HPLC separation of natural enantiomers **1** and **2**.......................... 9

Figure S5. Chiral HPLC separation of natural enantiomers **3** and **4**......................... 10

Figure S6. Chiral HPLC separation of synthetic compounds **1–4**............................11

Experimental Section 12

General Experimental Procedures.. 12

Plant Materia.. 12

Extraction and Isolation of (–) and (+)-Securidanes A and B (1–4)............ 12

PTP1B inhibition assay.. 14

Total Synthesis of (–) and (+)-Securidanes A and B (1–4)............................ 14

Synthesis of compound 11... 14

Synthesis of compound 10... 14

Synthesis of compound 7... 15

Synthesis of compound 6... 16

Synthesis of compound 6a... 16

Synthesis of compounds 1a–4a... 17

Synthesis of compounds 1–4... 18

Figure S7. Docking poses of compounds **1–4** (a–d) at the active site of PTP1B. 19

Figure S8. Key interactions between the docked compounds and PTP1B analyzed by Ligplot+ 20

X-ray Crystal Data for (–)-Securidane A (1) 21

X-ray Crystal Data for (+)-Securidane A (2) 22

Figure S9. ^{1}H NMR spectrum of (–)-securidane A (**1**) in $CDCl_3$............................. 24

Figure S10. ^{13}C NMR spectrum of (–)-securidane A (**1**) in $CDCl_3$ 25
Figure S11. ^{1}H–^{1}H COSY spectrum of (–)-securidane A (**1**) in $CDCl_3$ 26
Figure S12. HSQC spectrum of (–)-securidane A (**1**) in $CDCl_3$ 27
Figure S13. HMBC spectrum of (–)-securidane A (**1**) in $CDCl_3$ 28
Figure S14. ESI(+)MS spectrum of (–)-securidane A (**1**) 29
Figure S15. ESI(–)MS spectrum of (–)-securidane A (**1**) 30
Figure S16. HRESI(+)MS spectrum of (–)-securidane A (**1**) 31
Figure S17. IR spectrum of (–)-securidane A (**1**) 32
Figure S18. ^{1}H NMR spectrum of (+)-securidane A (**2**) in $CDCl_3$ 33
Figure S19. ^{13}C NMR spectrum of (+)-securidane A (**2**) in $CDCl_3$ 34
Figure S20. ^{1}H NMR spectrum of (–)-securidane B (**3**) in $CDCl_3$ 35
Figure S21. ^{13}C NMR spectrum of (–)-securidane B (**3**) in $CDCl_3$ 36
Figure S22. ^{1}H–^{1}H COSY spectrum of (–)-securidane B (**3**) in $CDCl_3$ 37
Figure S23. HSQC spectrum of (–)-securidane B (**3**) in $CDCl_3$ 38
Figure S24. HMBC spectrum of (–)-securidane B (**3**) in $CDCl_3$ 39
Figure S25. ESI(+)MS spectrum of (–)-securidane B (**3**) 40
Figure S26. ESI(–)MS spectrum of (–)-securidane B (**3**) 41
Figure S27. HRESI(+)MS spectrum of (–)-securidane B (**3**) 42
Figure S28. IR spectrum of (–)-securidane B (**3**)... 43
Figure S29. ^{1}H NMR spectrum of (+)-securidane B (**4**) in $CDCl_3$ 44
Figure S30. ^{13}C NMR spectrum of (+)-securidane B (**4**) in $CDCl_3$ 45
Figure S31. ^{1}H NMR spectrum of compound **11** in $CDCl_3$ 46
Figure S32. ^{13}C NMR spectrum of compound **11** in $CDCl_3$ 47
Figure S33. ^{1}H NMR spectrum of compound **10** in $CDCl_3$ 48
Figure S34. ^{13}C NMR spectrum of compound **10** in $CDCl_3$ 49
Figure S35. ^{1}H NMR spectrum of compound **7** in $CDCl_3$ 50
Figure S36. ^{13}C NMR spectrum of compound **7** in $CDCl_3$ 51
Figure S37. ^{1}H NMR spectrum of compound **6** in $CDCl_3$ 52
Figure S38. ^{13}C NMR spectrum of compound **6** in $CDCl_3$ 53
Figure S39. ^{1}H NMR spectrum of compound **6a** in $CDCl_3$ 54

Figure S40. ^{13}C NMR spectrum of compound **6a** in $CDCl_3$..................................... 55
Figure S41. ^{1}H NMR spectrum of synthetic compounds **3a** + **4a** (with minor **1a** + **2a**) in $CDCl_3$ 56
Figure S42. ^{13}C NMR spectrum of synthetic compounds **3a** + **4a** (with minor **1a** + **2a**) in $CDCl_3$ 57
Figure S43. LC-MS of **1** (**a**), **3** (**b**) and the ethanolic crude extract (**c**)..................... 58

Table S1. ^{1}H (500 MHz) and ^{13}C NMR (125 MHz) Data of **1–4** in $CDCl_3$

	**1(2)**		**3(4)**	
Position	δ_H	δ_C	δ_H	δ_C
1		128.2		127.8
2		141.6		141.7
3		137.5		137.1
4		148.8		148.6
5	6.46, d (8.4)	102.9	6.50, d (8.1)	103.2
6	6.43, d (8.4)	122.1	6.64, d (8.1)	122.6
7		145.0		141.8
8, 12	7.06, m	128.5	7.21, m	128.3
9, 11	7.10, m	129.0	7.31, m	128.7
10	7.24, m	127.0	7.24, m	126.6
13	5.58, s	45.0	6.30, s	40.4
1′		142.1		141.4
2′	6.45 d (2.8)	108.8	6.75, d (1.7)	109.3
3′		159.0		156.1
4′	6.44 d (2.8)	102.3		116.7
5′		156.4		158.3
6′		119.4	6.73, d (1.7)	102.4
7′		142.2		140.9
8′, 12′	7.29, m	127.8	7.59, m	126.9
9′, 11′	7.28, m	128.9	7.42, m	128.7
10′	7.22, m	126.8	7.33, m	127.4
-OCH_2O-	5.93, d (1.4)	101.2	5.96, d (1.5)	101.3
	5.95, d (1.4)		5.97, d (1.5)	
2-OCH_3	3.50, s	59.2	3.74, s	59.6
3′-OCH_3	3.78, s	55.3	3.83, s	56.2
5′-OH	5.40, s		5.64, s	

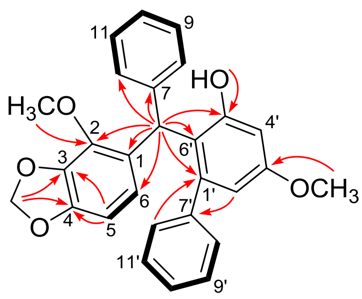

Figure S1. ^{1}H–H COSY (bold bond) and key HMBC (red arrows) correlations of **1**.

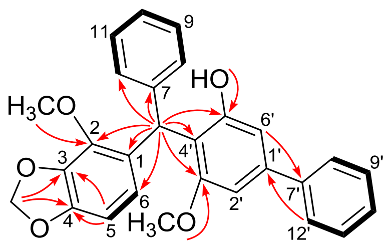

Figure S2.^{1}H–H COSY (bold bond) and key HMBC (red arrows) correlation of **3**

Figure S3. HPLC separation of the natural enantiomeric pairs (**1** + **2**) and (**3** + **4**)

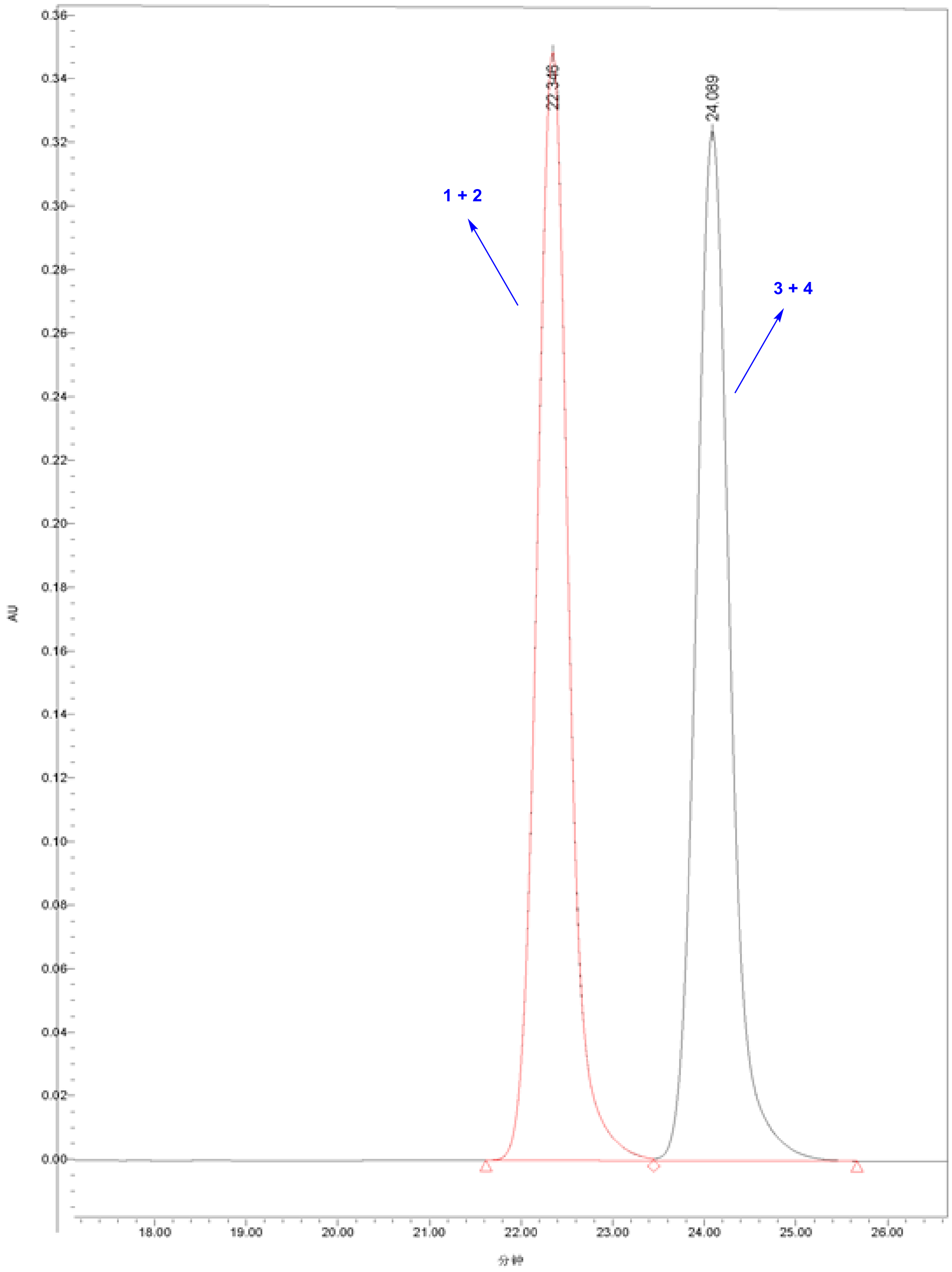

* YMC-Pack ODS-A (250 mm × 10 mm, S-5 μm) column
Mobile Phase: MeOH/H_2O 4:1, v/v, 3 ml min^{-1}

compound	retention time (min)	peak area (%)
**1 + 2**	22.346	50.1
**3 + 4**	24.089	49.9

Figure S4. Chiral HPLC separation of natural enantiomers **1** and **2**

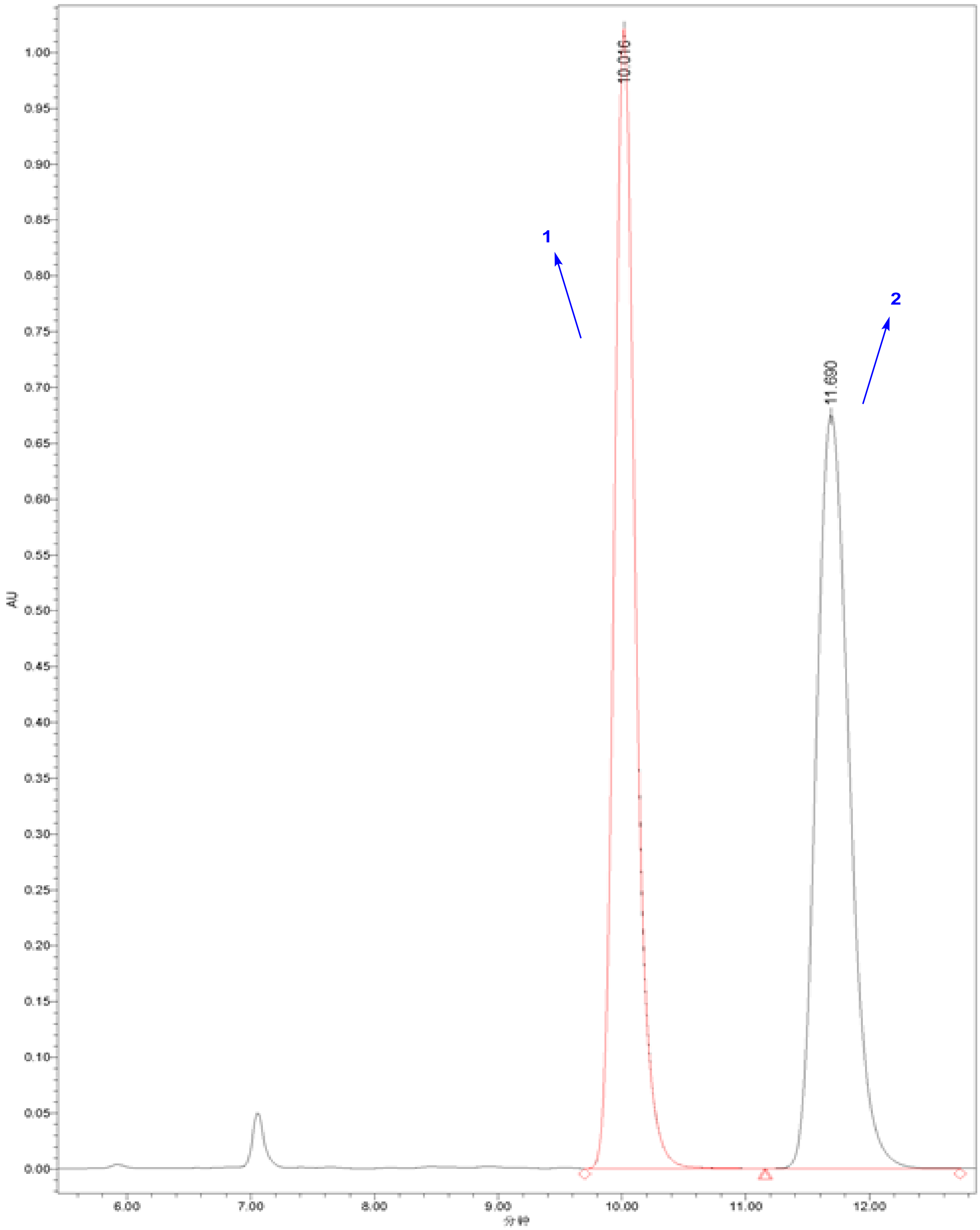

∗ Daicel CHIRALPAK AD-H (250 mm × 10 mm, S-5 μm) column
Mobile Phase: n-hexane/isopropanol 9:1, v/v, 3 ml min^{-1}

compound	retention time (min)	peak area (%)
**1**	10.016	49.25
**2**	11.690	50.75

Figure S5. Chiral HPLC separation of natural enantiomers **3** and **4**

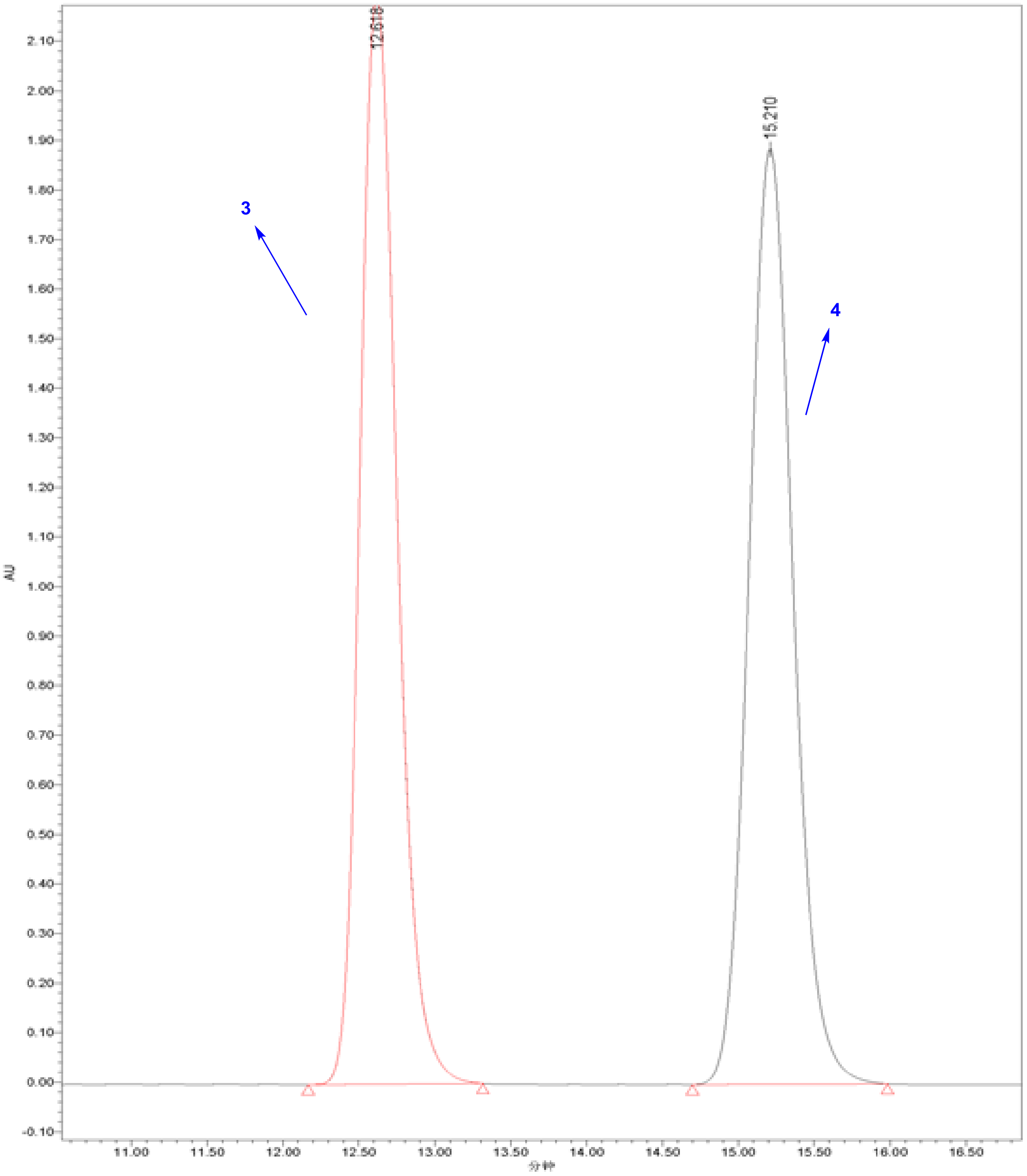

∗ Daicel CHIRALPAK AD-H (250 mm × 10 mm, S-5 μm) column
Mobile Phase: n-hexane/isopropanol 4:1, v/v, 3 ml min^{-1}

compound	retention time (min)	peak area (%)
**3**	12.618	49.25
**4**	15.210	50.75

Figure S6. Chiral HPLC separation of synthetic compounds **1–4**

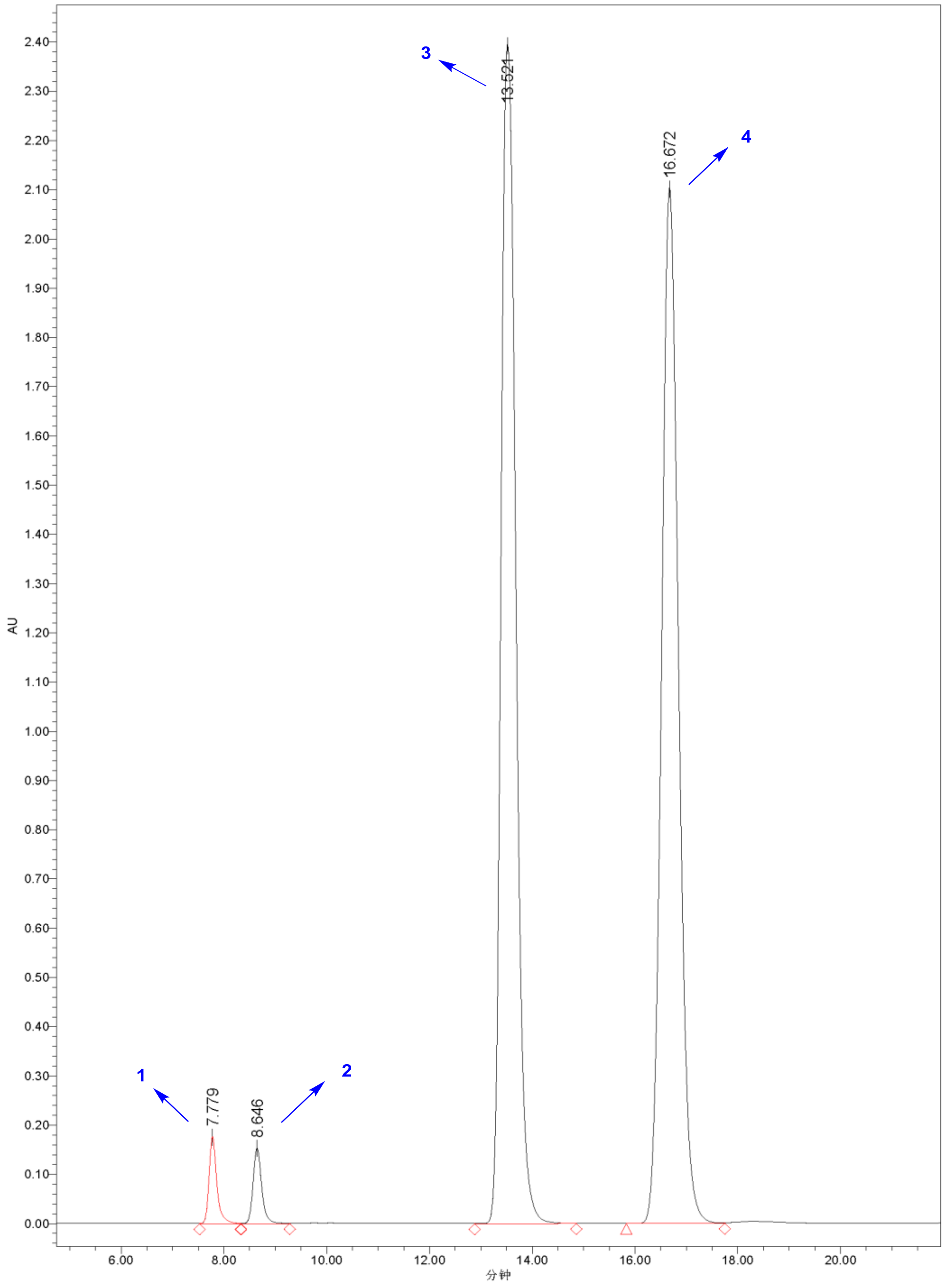

∗ Daicel CHIRALPAK AD-H (250 mm × 10 mm, S-5 μm) column
Mobile Phase: n-hexane/isopropanol 5:1, v/v, 3 ml min^{-1}

compound	retention time (min)	peak area (%)
**1**	7.779	1.86
**2**	8.646	1.80
**3**	13.521	47.92
**4**	16.672	48.42

Experimental Section

General Experimental Procedures

Optical rotations were measured on an Autopol VI polarimeter at room temperature. UV data were obtained by using a Shimadzu UV-2550 spectrophotometer. IR spectra were acquired on a Thermo IS5 or a Nicolet NEXUS 670 FT-IR spectrometer with KBr disks. NMR spectra were collected on a Bruker AM-500 or AM-400 NMR spectrometer with TMS as internal standard. ESIMS and HRESIMS were performed on a Brucker Daltonics Esquire 3000 plus LCMS and a Waters-Micromass Q-TQF Ultima Global mass spectrometer (or a Bruker Apex II mass spectrometer), respectively. Semipreparative HPLC was performed on a Waters 1525 binary pump system with a Waters 2489 detector (210 nm) and equipped with a YMC-Pack ODS-A (250 mm × 10 mm, S-5 *μ*m) or a Daicel CHIRALPAK AD-H (250 mm × 10 mm, S-5 μm) column. Silica gel (200–300 mesh, Qingdao Haiyang Chemical Co., Ltd), C18 reversed-phase (RP-18) silica gel (20–45 *μ*M, Fuji Silysia Chemical Ltd.), CHP20P MCI gel (75–150 *μ*m, Mitsubishi Chemical Corporation), and Sephadex LH-20 gel (Amersham Biosciences) were used for column chromatography (CC). Pre-coated silica gel GF254 plates (Qingdao Haiyang Chemical Co., Ltd.) were used for TLC detection. All solvents used for CC were of analytical grade (Shanghai Chemical Reagents Co., Ltd.), and solvents used for HPLC were of HPLC grade (J & K Scientific Ltd.).

Plant Material

The stem of *S. inappendiculata* were collected from Guilin of Guangxi Province, People's Republic of China, and were authenticated by Professor Shao-Qing Tang of Guangxi Normal University. A voucher specimen has been deposited in Shanghai Institute of Materia Medica, Chinese Academy of Sciences (accession number: Sinap-2011-01Y)

Extraction and Isolation of (–) and (+)-Securidanes A and B (1–4)

The stem powder of *S. inappendiculata* (2.5 kg) were extracted with 95% EtOH (3 ×

10 L) at room temperature to give a crude extract (150 g), which was then partitioned between EtOAc and H_2O. The EtOAc soluble fraction (50 g) was separated by a CHP20P MCI gel column ($MeOH/H_2O$, 4:6 to 9:1) to afford three fractions A–C. Fraction B (6.2 g) was separated on a silica gel column and eluted with gradient mixtures of petroleum ether-acetone (from 50:1 to 1:5) to afford six fractions (B1–B6). B1 (1.2 g) was purified with semipreparative HPLC (80% CH_3CN in H_2O as the mobile phase, 3 mL/min) to give **5** (45 mg)**.** Fraction B3 (2.7 g) was separated on a column of reversed phase C_{18} silica gel (80% aqueous methanol, v/v), and then semipreparative HPLC (85% CH_3CN in H_2O, 3 mL/min) to give two enantiomeric mixtures M1 (**1** and **2)** and M2 **(3** and **4)**. The two enantiomeric mixtures M1 and M2 were finally resolved by chiral separation on semipreparative HPLC equipped with a Daicel CHIRALPAK AD-H column to give compounds **1** (14.2 mg) and **2** (14.8 mg) (mobile phase: n-hexane/isopropanol 9:1, v/v, 3 mL/min), and compounds **3** (13.7 mg) and **4** (14.7 mg) (mobile phase: n-hexane/isopropanol 4:1, v/v, 3 mL/min), respectively.

(–)-Securidane A (**1**): colorless crystals (MeOH); $[\alpha]^{22}_{D}$ –76.9 (*c* 0.42, MeOH); IR (KBr) ν_{max} 3487, 2921, 1613, 1576, 1467, 1345, 1256, 1208, 1162, 1073, 1044, 980, 695 cm^{-1}; ^{1}H and ^{13}C NMR ($CDCl_3$), see Table S1; (+)-ESIMS *m/z* 440.9 $[M + H]^+$; (–)-ESIMS *m/z* 439.1 $[M - H]^-$; (+)-HRESIMS *m/z* 441.1700 $[M + H]^+$ (calcd for $C_{28}H_{25}O_5$, 441.1697).

(+)-Securidane A (**2**): colorless crystals (MeOH); $[\alpha]^{22}_{D}$ +72.3 (*c* 0.48, MeOH).

(–)-Securidane B (**3**): colorless gum; $[\alpha]^{22}_{D}$ –43.5 (*c* 0.37, MeOH); IR (KBr) ν_{max} 3488, 2934, 2844, 1614, 1565, 1467, 1409, 1255, 1224, 1094, 1067, 1042, 765, 699 cm^{-1}; ^{1}H and ^{13}C NMR ($CDCl_3$), see Table S1; (+)-ESIMS *m/z* 441.0 $[M + H]^+$, 903.0 $[2 M + Na]^+$; (–)-ESIMS *m/z* 439.1 $[M - H]^-$; (+)-HRESIMS *m/z* 441.1707 $[M + H]^+$ (calcd for $C_{28}H_{25}O_5$, 441.1697).

(+)-Securidane B (**4**): colorless gum; $[\alpha]^{22}_{D}$ +42.3 (*c* 0.47, MeOH).

PTP1B inhibition assay: A colorimetric assay to measure inhibition against PTP1B was performed same as reported 96-well plates. Briefly, the tested compounds were solubilized in DMSO and serially diluted into concentrations for the inhibitory test. The assays were carried out in a final volume of 100 μL containing 50 mmol/L MOPS, pH 6.5, 2 mmol/L pNPP, 30 nmol/L GST-PTP1B, and 2% DMSO, and the catalysis of pNPP was continuously monitored on a SpectraMax 340 microplate reader at 405 nm for 3 min at 30 ℃. The IC_{50} value was calculated from the nonlinear curve fitting of the percent inhibition [inhibition (%)] vs the inhibitor concentration [I] using the following equation: %inhibition=100/{1+(IC_{50}/[I]) *k*}, where *k* is the Hill coefficient.

Total Synthesis of (–) and (+)-Securidanes A and B (1–4)

Synthesis of compound 11

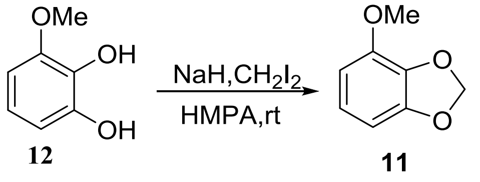

Sodium hydride (1.71 g, 42 mmol) followed by diiodomethane (2.1 mL, 26 mmol) was added to a solution of 3-methoxypyrocatechol (3.0 g, 21 mmol) in hexamethylphosphoramide (HMPA) (75 mL). The reaction was kept at 50 ℃ for 6 h. After cooling down to room temperature, the reaction mixture was poured into ice water (350 mL) and then extracted with ether (3 x 75 mL). The organic phase was washed with brine and dried with anhydrous Na_2SO_4. After work up, the crude product was purified by a flash column over silica gel (Petroleum Ether/EtOAc = 32:1) to give compound **11** as a white solid (2.9 g, 87%). ^{1}H NMR ($CDCl_3$, 400 MHz): δ 6.78 (t, 1H, J = 8.2 Hz) , 6.53 (d, 2H, J = 8.2 Hz), 5.95 (s, 2 H), 3.90 (s, 3 H); ^{13}C NMR ($CDCl_3$, 100 MHz): δ 148.9, 144.2, 135.3, 122.1, 107.6, 102.5, 101.2, 56.6; IR(KBr) V_{max} 3006, 2894, 1639, 1503, 1463, 1287, 1254, 1092, 1056, 963, 927, 758, 711 cm^{-1}; (+)-HRESIMS *m/z* 153.0545 $[M + H]^+$ (calcd for $C_8H_9O_3$, 153.0546).

Synthesis of compound 10

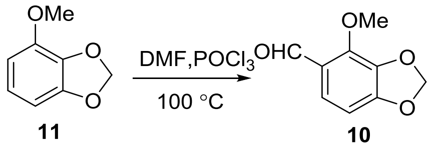

Phosphorus oxychloride (3.7 mL, 40.7 mmol) was added dropwise at room temperature to a solution of compound **11** (2.48 g, 16.3 mmol) in dimethylformamide (DMF, 5 mL) and was stirred for 30 min. The reaction was heated to 100 ℃ and kept for 7 h. After cooled down, the reaction mixture was poured into 25% sodium acetate solution (100 mL) and stirred for 30 min. The resultant solution was extracted with EtOAc and the organic phase was then washed with saturated sodium bicarbonate, water and brine in turn, and dried with anhydrous Na_2SO_4. After work up, the crude product was purified by flash column chromatography over silica gel (Petroleum Ether/EtOAc = 32:1) to obtain compound **10** as a white solid (1.53 g, 52%). ^{1}H NMR ($CDCl_3$, 400 MHz): δ 10.21 (s, 1H), 7.45 (d, 1H, J = 8.2 Hz), 6.59 (d, 1H, J = 8.2 Hz), 6.03 (s, 2H), 4.12 (s, 3H); ^{13}C NMR ($CDCl_3$, 100 MHz): δ 188.3, 154.8, 146.3, 136.0, 124.4, 123.0, 103.4, 102.0, 60.3; IR (KBr): V_{max} 3400, 2915, 1736, 1710, 1665, 1483, 1470, 1348, 1277, 1242, 1076, 1041, 945, 805, 787 cm^{-1}; (+)-HRESIMS *m/z* 181.0493 $[M + H]^+$ (calcd for $C_9H_9O_4$, 181.0495).

Synthesis of compound 7

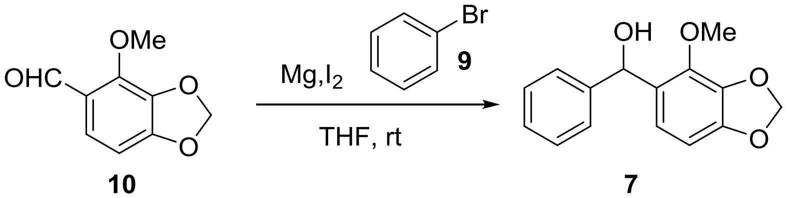

A solution of iodine (0.2 mL, 2.3 mmol) in THF (3 mL) was added to a 150 mL three necked flask containing Mg turnings (1.3 g, 54.0 mmol) under nitrogen atmosphere, and stirred for a few minutes. A solution of bromobenzene (6.3 mL, 54.0 mmol) in THF (40 mL) was then added dropwise. The suspension was stirred for an additional 30 min at room temperature, and a solution of **10** (1.96 g, 10.8 mmol) in THF (20 mL) was added slowly, and the reaction was stirred for 6 h. The resultant mixture was quenched with aq. NH_4Cl and extracted with ethyl acetate. The organic phase was dried with anhydrous Na_2SO_4. After removal of solvent under vacuum, the crude product was purified by column chromatography over silica gel (petroleum ether/EtOAc = 32:1) to give the secondary benzylic alcohol (**7**) (2.8 g, 99%). ^{1}H NMR

($CDCl_3$, 400 MHz): δ 7.40 (m, 2H), 7.36 (m, 1H), 7.28 (m, 1H), 6.80 (d, 1H, J = 8.1 Hz), 6.55 (d, 1H, J = 8.0 Hz), 5.96 (s, 1H), 5.94 (d, 1H, J = 1.5 Hz), 5.93 (d, 1H, J = 1.5 Hz), 3.86 (s, 3H), ^{13}C NMR ($CDCl_3$, 100 MHz): δ 149.1, 144.0, 141.1, 136.7, 129.5, 128.2, 127.2, 126.4, 120.8, 102.6, 101.2, 72.4, 59.6; IR (KBr): V_{max} 3544, 3419, 3061, 2891, 1629, 1608, 1469, 1259, 1067, 1035, 923, 792, 758, 701 cm^{-1}. (+)-HRESIMS m/z 1241.0856 [M – H_2O + H]$^+$ (calcd for $C_{15}H_{13}O_{34}$, 241.0859).

Synthesis of compound 6

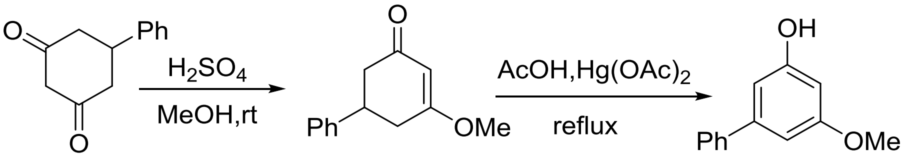

8 8a 6

To 50 mL methanol solution of phenylcyclohexadienone (2.5 g, 13.3 mmol), sulfuric acid (0.30 g) was added and the solution was stirred at room temperature for about 9 h. After removal of methanol the reaction mixture was dissolved in about 50mL DCM, and washed with a saturated sodium bicarbonate solution and brine successively, and dried with anhydrous Na_2SO_4. Evaporation of the solvent gave 2.5 g of 3-methoxy-5-phenyl-cyclohexenone (**8a**, yellow oil). To a solution of compound **8a** (2.5 g, 12.3 mmol) in 30 mL glacial acetic acid, mercury acetate (5.75 g, 18.0 mmol) was added and refluxed for 7h. After cooling down and filtration, the acetic acid solution was adjusted with aqueous sodium hydroxide solution to pH = 7, and extracted with ether. The organic phase was washed with brine and dried over anhydrous Na_2SO_4. Evaporation of the solvent gave a crude product, which was purified by a flash column over silica gel (petroleum Ether/EtOAc = 8:1) to give 5-methoxy-biphenyl-3-ol (**6**) as a pale oil (1.60 g, 60 %). ^{1}H NMR ($CDCl_3$, 400 MHz): δ 7.55 (m, 2H), 7.42 (m, 2H), 7.37 (m, 1H), 6.75 (t, 1H, J = 1.6 Hz), 6.68 (t, 1H, J = 1.6 Hz), 6.44 (t, 1H, J = 2.2 Hz), 5.47 (s, 1H), 3.84 (s, 3H). ^{13}C NMR ($CDCl_3$, 100 MHz): δ 161.2, 157.0, 143.9, 140.9, 128.8, 127.8, 127.2, 107.1, 105.9, 100.6, 55.6; IR (KBr): V_{max} 3390, 2960, 1614, 1597, 1487, 1422, 1349, 1192, 1153, 1051, 763, 733. 697 cm^{-1}. (+)-HRESIMS m/z 201.0906 [M + H]$^+$ (calcd for $C_{13}H_{13}O_2$, 201.0910).

Synthesis of compound 6a

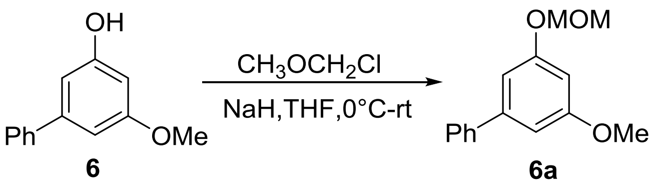

To a solution of **6** (0.28 g, 1.4 mmol) in DMF (30 mL) at 0 ℃, a suspension of NaH (1.2 equiv, 1.7 mmol) in DMF (5 mL) was added. The reaction was warmed to room temperature and stirred for 15 min. The reaction mixture was then cooled down to 0 ℃, and chloromethyl methyl ether (0.13 ml, 1.7 mmol) was added. The resulting suspension was warmed to room temperature and stirred overnight. The reaction suspension was then diluted with 50 mL of ether and poured into 50 mL water. The organic phase was washed with 1N KOH aq. (50 mL), H_2O (50 mL) and brine (50 mL), respectively, and dried over anhydrous Na_2SO_4. After workup, the product was purified by column chromatography over silica gel (petroleum ether/EtOAc = 8:1) to afford compound **6a** (0.336 g, 98%). ^{1}H NMR ($CDCl_3$, 400 MHz): δ 7.59 (m, 2H), 7.44 (m, 2H), 7.37 (m, 1H), 6.91 (t, 1H, J = 1.8 Hz), 6.82 (q, 1H, J = 1.2 Hz), 6.64 (t, 1H, J = 2.2 Hz), 5.23 (s, 2H), 3.86 (s, 3H), 3.52 (s, 3H); ^{13}C NMR ($CDCl_3$, 100 MHz): δ 161.1, 158.8, 143.6, 141.1, 128.8, 127.5, 127.3, 107.8, 106.8, 101.5, 94.7, 56.2, 55.6. IR (KBr): V_{max} 3399, 2955, 2935, 1596, 1465, 1423, 1214, 1148, 1055, 1014, 928, 763, 699 cm^{-1}. (+)-HRESIMS m/z 245.1168 $[M + H]^+$ (calcd for $C_{15}H_{15}O_3$, 245.1172).

Synthesis of compounds 1a–4a

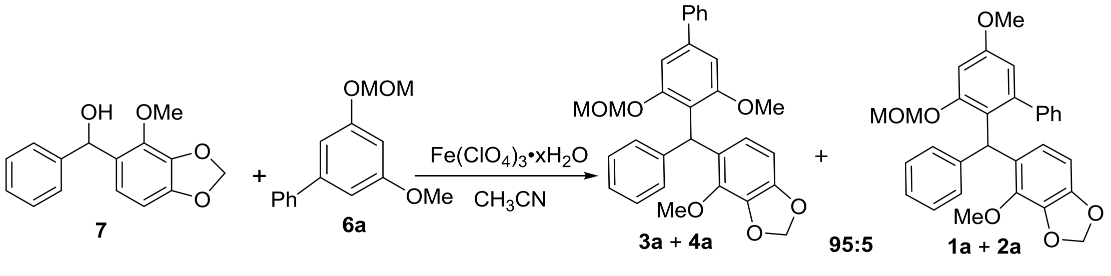

$Fe(ClO_4)_3{\bullet}H_2O$ (0.124g, 0.35 mmol) was added to a mixture of secondary benzyl alcohol (**7**, 1.79 g. 6.9 mmol) and arene (**6a**, 2.04 g, 8.3 mmol) in CH_3CN (5 mL). The resulting solution was stirred for 7h at 60 ℃. The reaction was quenched by adding water (10 mL) and extracted with ethyl acetate (10 mL). The organic phase was washed with water (10 mL) and aq. NH_4Cl (10 mL) in turn, and dried over anhydrous

Na_2SO_4. After removal of the solvent, the crude product was purified by a flash column packing with silica gel (petroleum ether/EtOAc = 32:1) to obtain a mixture (1.92 g, 57 %) of (**1a** + **2a**, minor, <5% as estimated by peak integration of H-13 at δ_H 5.60**)** and (**3a** + **4a**, major, >95% as estimated by peak integration of H-13 at δ_H 6.38). The NMR spectra of **3a** + **4a** (with minor **1a** + **2a**): ^{1}H NMR ($CDCl_3$, 400 MHz): δ 7.61 (d, 2H, *J* = 7.2 Hz), 7.44 (t, 2H, *J* = 7.4 Hz), 7.33 (d, 1H, *J* = 7.2 Hz), 7.23 (t, 2H, *J* = 7.2 Hz), 7.11–7.14 (t, 3H, *J* = 7.2 Hz), 7.02 (d, 1H, *J* = 1.2 Hz), 6.85 (s, 1H), 6.68 (d, 1H, *J* = 8.2 Hz), 6.49 (d, 1H, *J* = 8.2 Hz), 6.38 (s, 1H), 5.93 (dd, 2H, *J* = 9.6 Hz, *J* = 1.2 Hz), 4.93 (d, 1H, *J* = 6.9 Hz), 4.91 (d, 1H, *J* = 6.9 Hz), 3.74 (s, 3H), 3.70 (s, 3H), 3.25 (s, 3H). ^{13}C NMR ($CDCl_3$, 100 MHz): δ 159.0, 156.8, 147.7, 144.5, 142.2, 141.4, 141.3, 137.2, 129.2, 128.8 × 4, 127.6 × 2, 127.5, 127.2 × 2, 125.3, 123.5, 121.1, 107.3, 105.1, 102.4, 101.0, 94.7, 59.6, 56.2, 55.9, 40.0. IR (KBr): V_{max} 3373, 2936, 2898, 2369, 1599, 1567, 1467, 1256, 1153, 1112, 1068, 910, 732, 700 cm^{-1}. (+)-HRESIMS *m/z* 485.1954 $[M + H]^+$ (calcd for $C_{30}H_{29}O_6$, 485.1959).

Synthesis of compounds 1–4

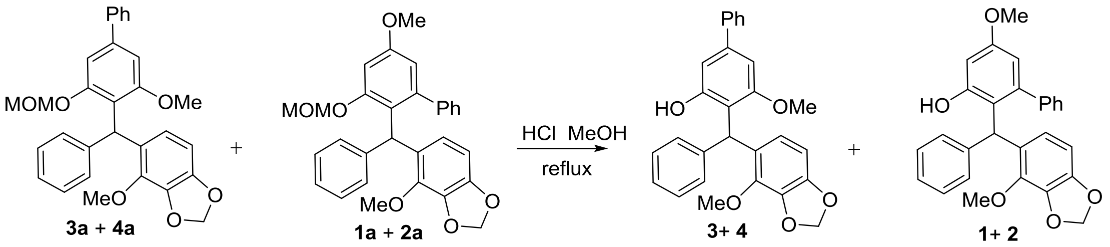

To a stirred solution of the mixture of compounds **1a–4a** (500 mg, 1.0 mmol) in MeOH (10 mL), 2N HCl (5.0 mL) was added and refluxed for 4 h. After removal solvents under vacuum, the resulting mixture was extracted with EtOAc (3 × 50 mL), and the organic phase was washed with brine and dried over anhydrous Na_2SO_4. After workup, the crude product was separated by flash column chromatography over silica gel (petroleum ether/EtOAc = 16:1) to obtain a mixture of two pair enantiomers (**1** + **2**) and (**3** + **4**) (0.36 g, 79%), which was further separated by chiral HPLC (n-hexane/isopropanol 5:1, v/v, 3 ml min^{-1}) to afford four optically pure compounds **1–4**, respectively.

PTP1B Inhibitory Evaluation and Molecular Docking of Compounds 1–4

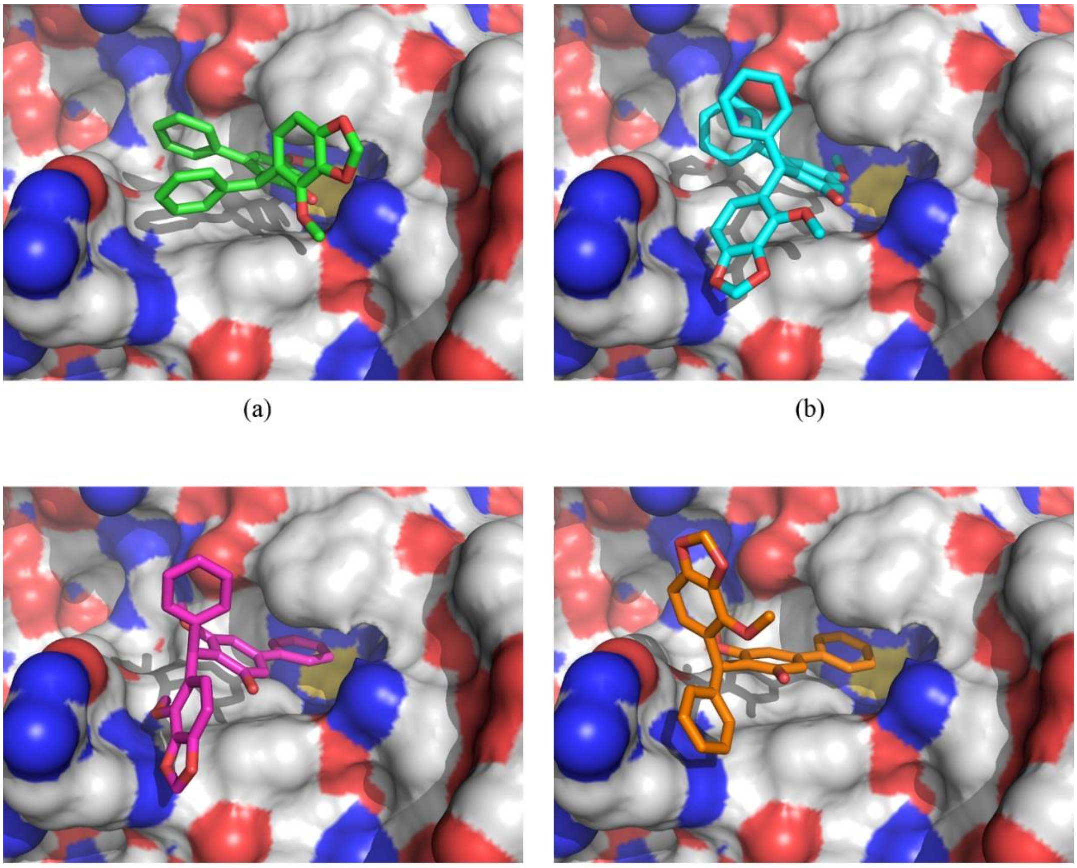

(a) (b) (c) (d)

Figure S7. Docking poses of compounds **1–4** (a–d) at the active site of PTP1B. Carbon atoms in compounds **1–4** are colored *green*, *cyan*, *magenta* and *orange*, respectively. Oxygen, nitrogen and sulfur atoms are colored *red*, *blue* and *yellow*, respectively. Compounds are represented as sticks. The protein is shown as white molecular surface.

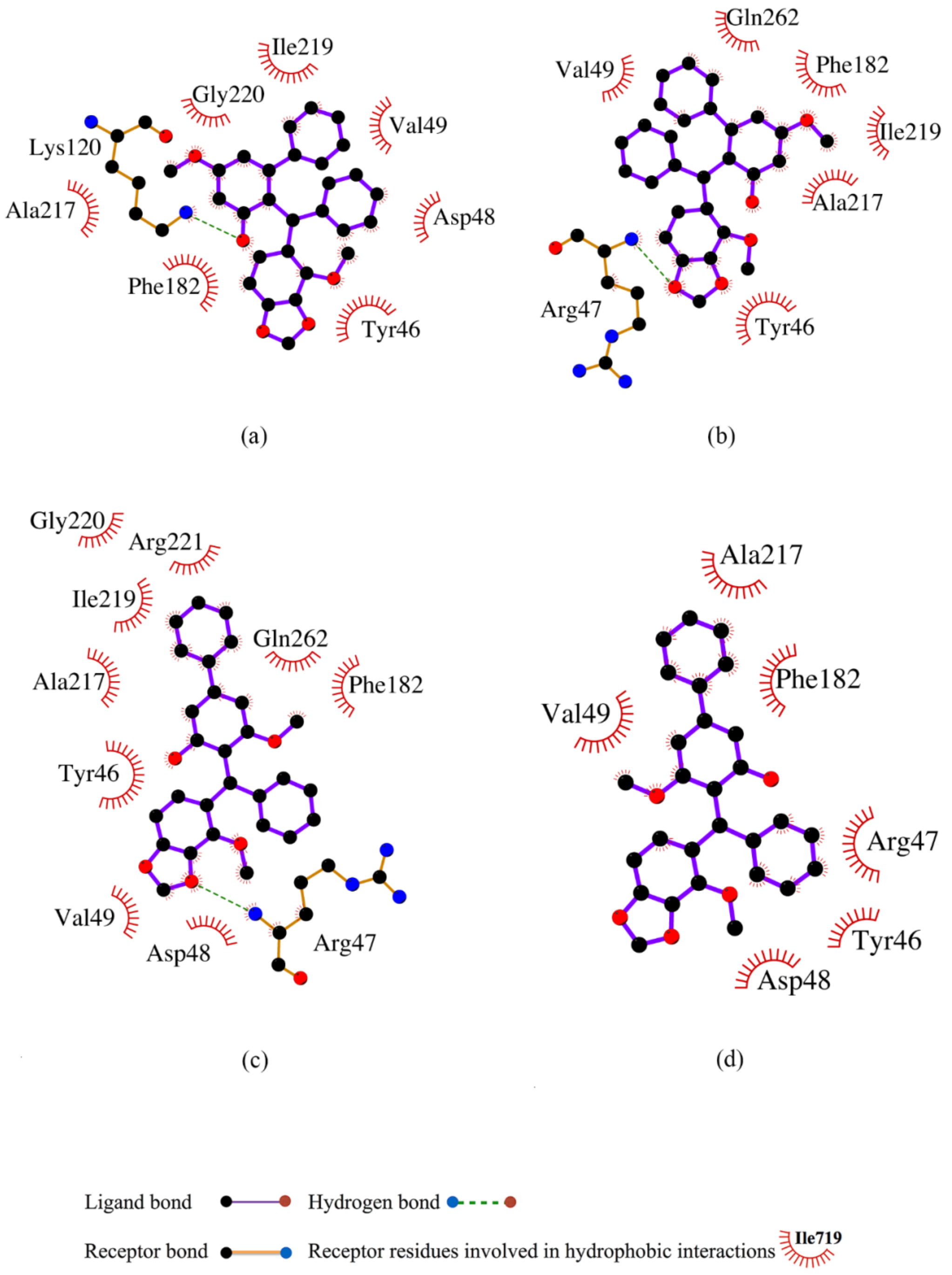

Figure S8. Key interactions between the docked compounds and PTP1B analyzed by Ligplot+

X-ray Crystal Data for (−)-Securidane A (1)

Identification code	cu_dm14282_0m	
Empirical formula	C28 H24 O5	
Formula weight	440.47	
Temperature	140(2) K	
Wavelength	1.54178 Å	
Crystal system	Orthorhombic	
Space group	P 21 21 21	
Unit cell dimensions	a = 6.94580(10) Å	α= 90 °.
	b = 10.10070(10) Å	β= 90 °.
	c = 31.6072(3) Å	γ = 90 °.
Volume	2217.48(4) $Å^3$	
Z	4	
Density (calculated)	1.319 Mg/m^3	
Absorption coefficient	0.731 mm^{-1}	
F(000)	928	
Crystal size	0.250 x 0.160 x 0.120 mm^3	
Theta range for data collection	2.796 to 69.478 °.	
Index ranges	-7<=h<=8, -12<=k<=9, -38<=l<=38	
Reflections collected	10852	
Independent reflections	4035 [R(int) = 0.0224]	
Completeness to theta = 67.679 °	99.3 %	
Absorption correction	Semi-empirical from equivalents	
Max. and min. transmission	0.7532 and 0.6106	
Refinement method	Full-matrix least-squares on F^2	
Data / restraints / parameters	4035 / 0 / 301	
Goodness-of-fit on F^2	1.033	
Final R indices [I>2sigma(I)]	R1 = 0.0343, wR2 = 0.0896	
R indices (all data)	R1 = 0.0356, wR2 = 0.0909	
Absolute structure parameter	-0.13(7)	
Extinction coefficient	n/a	
Largest diff. peak and hole	0.133 and -0.211 e.$Å^{-3}$	

X-ray Crystal Data for (+)-Securidane A (2)

Identification code	cu_dm15539_0m	
Empirical formula	C28 H24 O5	
Formula weight	440.47	
Temperature	296.15 K	
Wavelength	1.54178 Å	
Crystal system	Orthorhombic	
Space group	P 21 21 21	
Unit cell dimensions	a = 7.0002(8) Å	α= 90 °.
	b = 10.1031(13) Å	β= 90 °.
	c = 32.355(4) Å	γ = 90 °.
Volume	2288.3(5) Å^3	
Z	4	
Density (calculated)	1.279 Mg/m^3	
Absorption coefficient	0.708 mm^{-1}	
F(000)	928	
Crystal size	0.25 x 0.22 x 0.2 mm^3	
Theta range for data collection	2.731 to 69.548 °.	
Index ranges	-7<=h<=8, -12<=k<=12, -38<=l<=38	
Reflections collected	13506	
Independent reflections	4050 [R(int) = 0.0374]	
Completeness to theta = 67.679 °	99.5 %	
Absorption correction	Semi-empirical from equivalents	
Max. and min. transmission	0.7532 and 0.5837	
Refinement method	Full-matrix least-squares on F^2	
Data / restraints / parameters	4050 / 0 / 302	
Goodness-of-fit on F^2	1.044	
Final R indices [I>2sigma(I)]	R1 = 0.0364, wR2 = 0.0981	
R indices (all data)	R1 = 0.0396, wR2 = 0.1014	
Absolute structure parameter	0.04(10)	
Extinction coefficient	0.0061(6)	
Largest diff. peak and hole	0.114 and -0.141 e.Å^{-3}	

Colorless crystals of **1** and **2** were obtained in the solvent of MeOH. Crystal data were obtained on a Bruker APEX-II CCD detector employing graphite monochromated Cu-Kα radiation (λ = 1.54178 Å) at 140(2) K and operating in the φ-ω scan mode. The structures were solved by direct methods using SHELXS-97 (Sheldrick 2008) and refined with full-matrix leastsquares calculations on *F*2 using SHELX-97 (Sheldrick 2008). All non-hydrogen atoms were refined anisotropically. The hydrogen atom positions were geometrically idealized and allowed to ride on their parent atoms.[1,2]

Crystallographic data for **1** and **2** have been deposited at the Cambridge Crystallographic Data Center with the deposition number of CCDC 1434484 and CCDC 1474480. A copy of the data can be obtained free of charge via the Internet at www.ccdc.cam.ac.uk/conts/ retrieving.html or on application to the CCDC, 12 Union Road, Cambridge CB2 1EZ, UK [tel: (+44) 1223-336-033; e-mail: deposit@ccdc.cam.ac.uk].

References:

[1] G. M. Sheldrick, *SHELXS-97: Program for X-ray Crystal Structure Solution*; University of Göttingen: Göttingen, Germany, 1997.

[2] G. M Sheldrick,. *SHELX-97: Program for X-ray Crystal Structure Refinement*; University of Göttingen: Göttingen, Germany, 1997.

Figure S9. ^{1}H NMR spectrum of (−)-securidane A (**1**) in $CDCl_3$

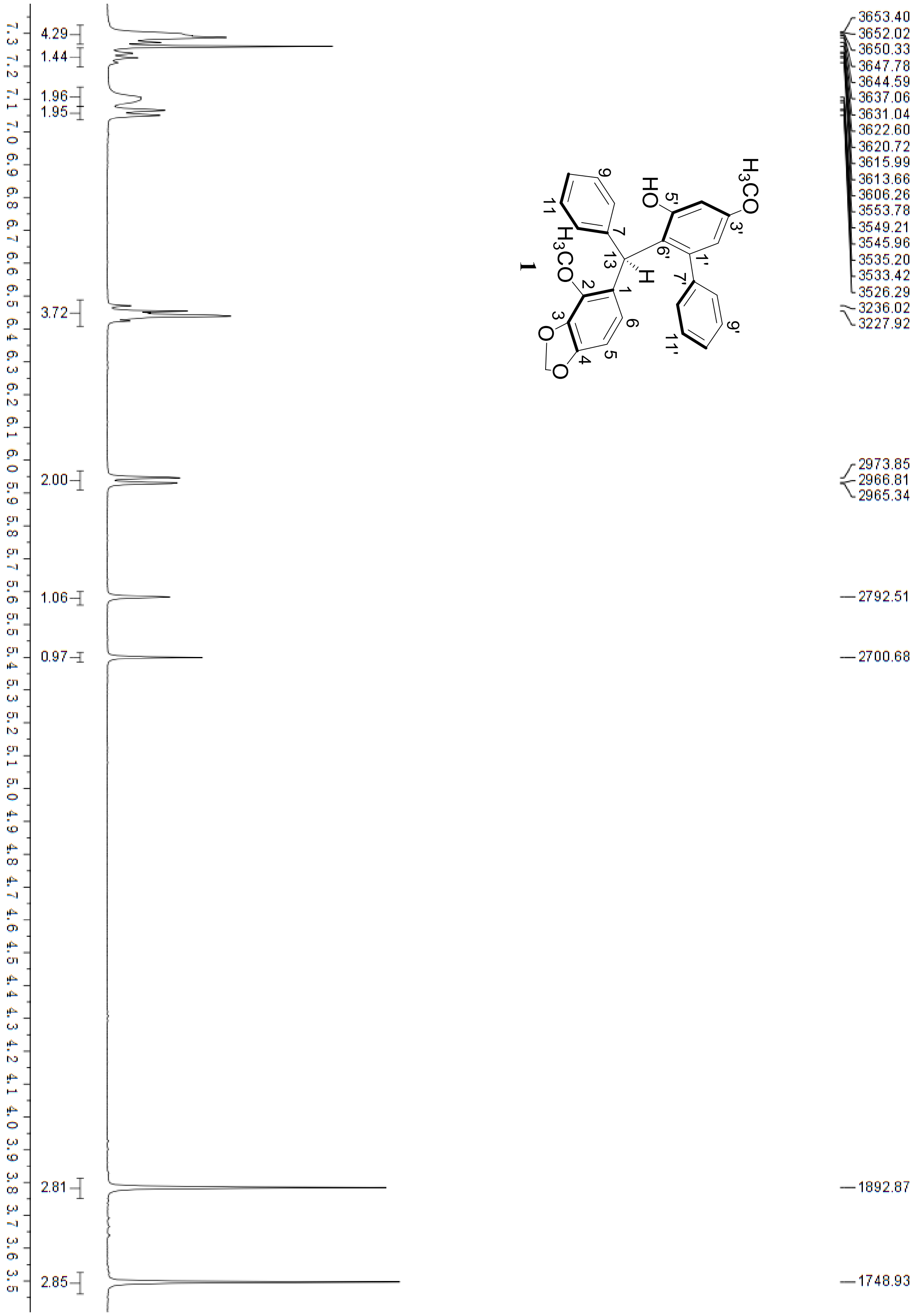

Figure S10. ^{13}C NMR spectrum of (–)-securidane A (**1**) in $CDCl_3$

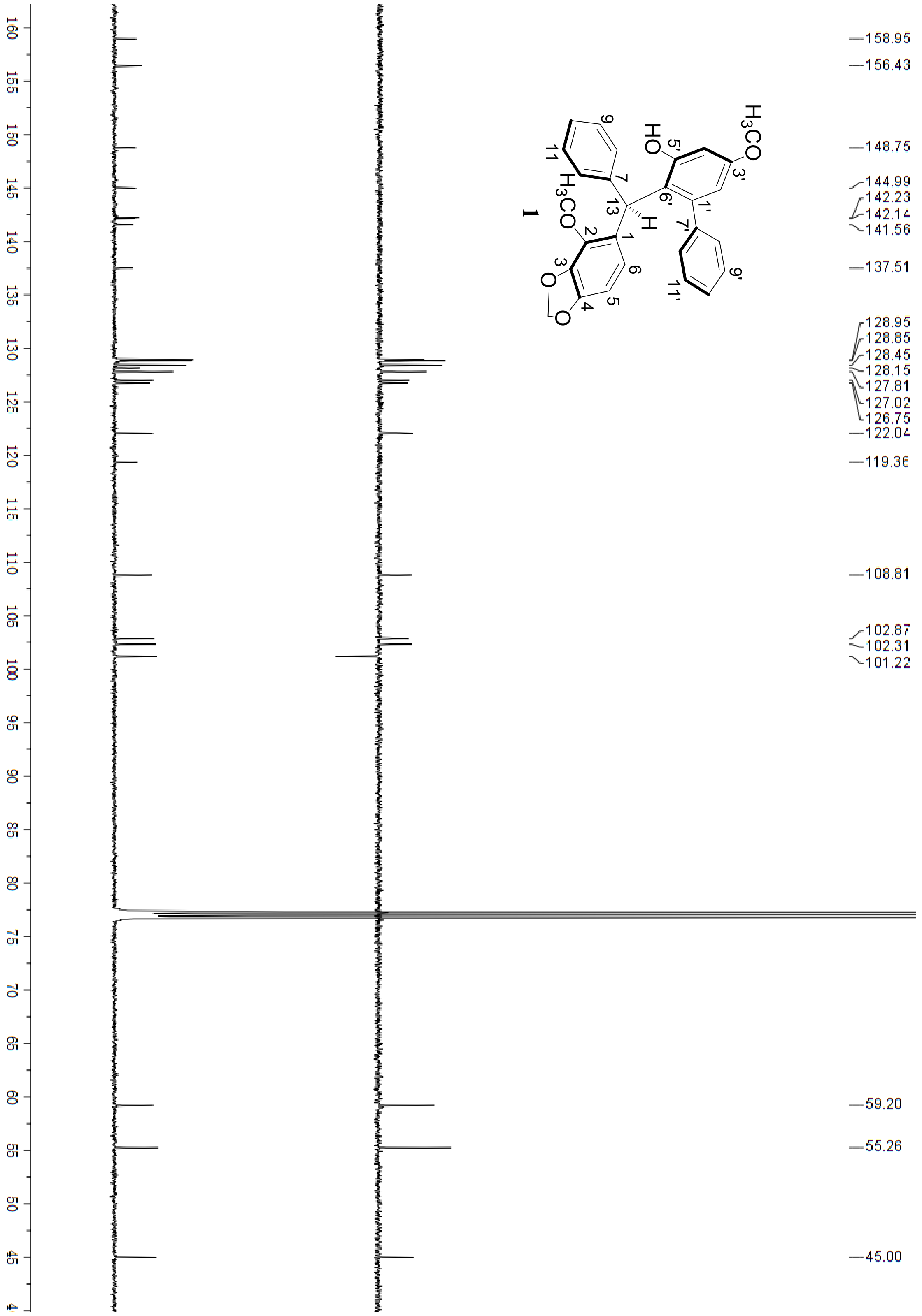

Figure S11. ^{1}H–^{1}H COSY spectrum of (–)-securidane A (**1**) in $CDCl_3$

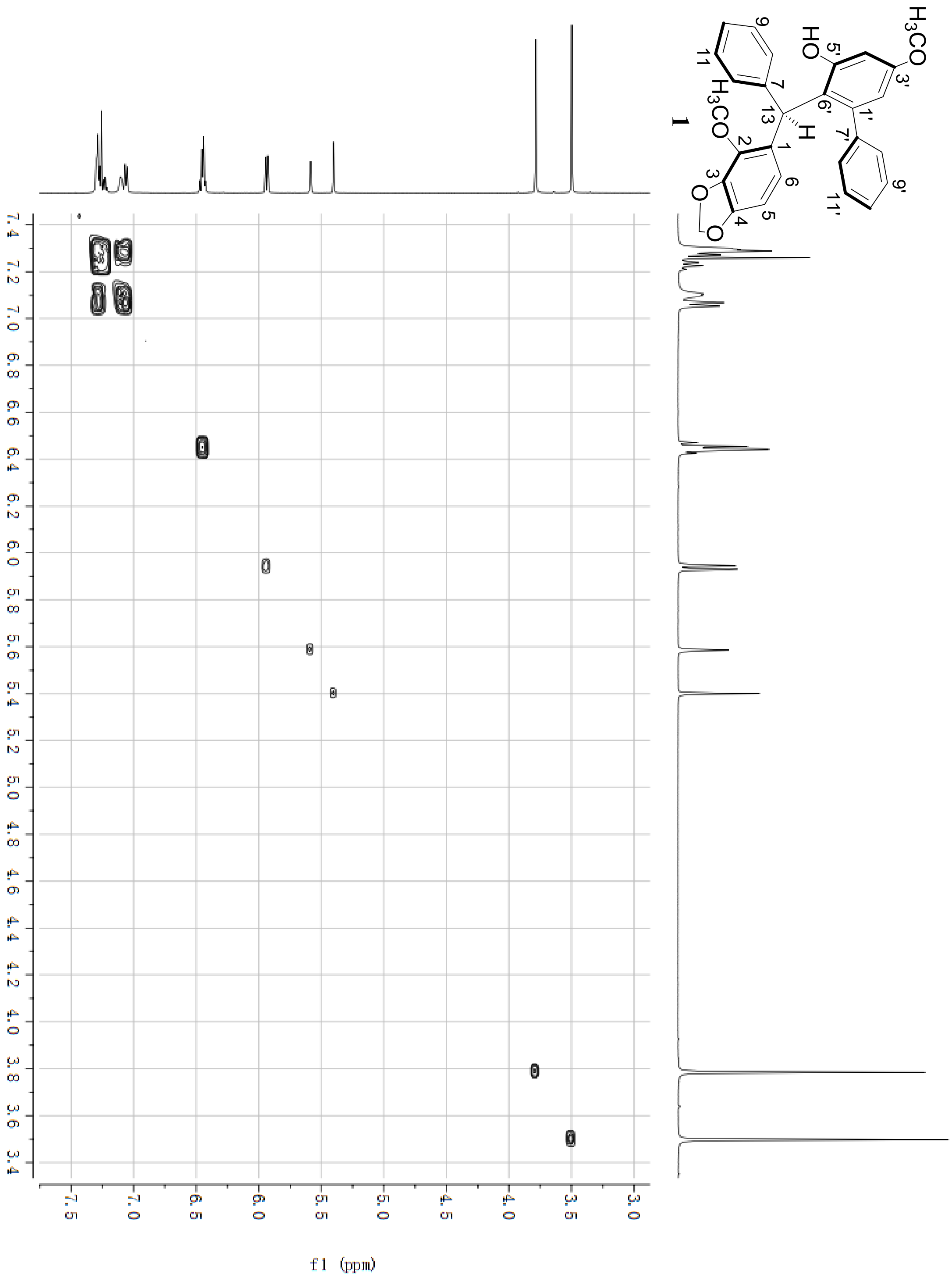

Figure S12. HSQC spectrum of (–)-securidane A (**1**) in $CDCl_3$

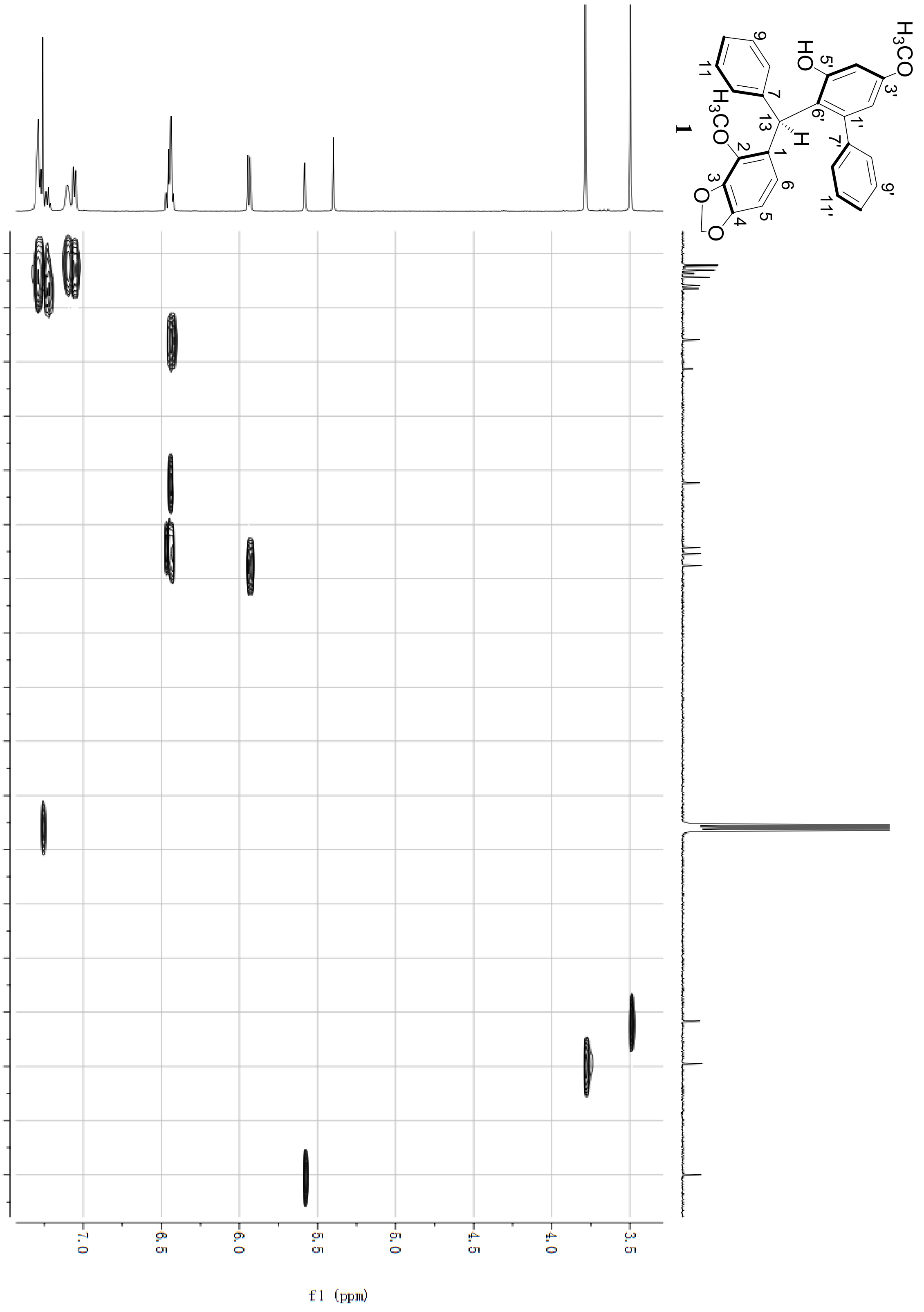

Figure S13. HMBC spectrum of (–)-securidane A (**1**) in $CDCl_3$

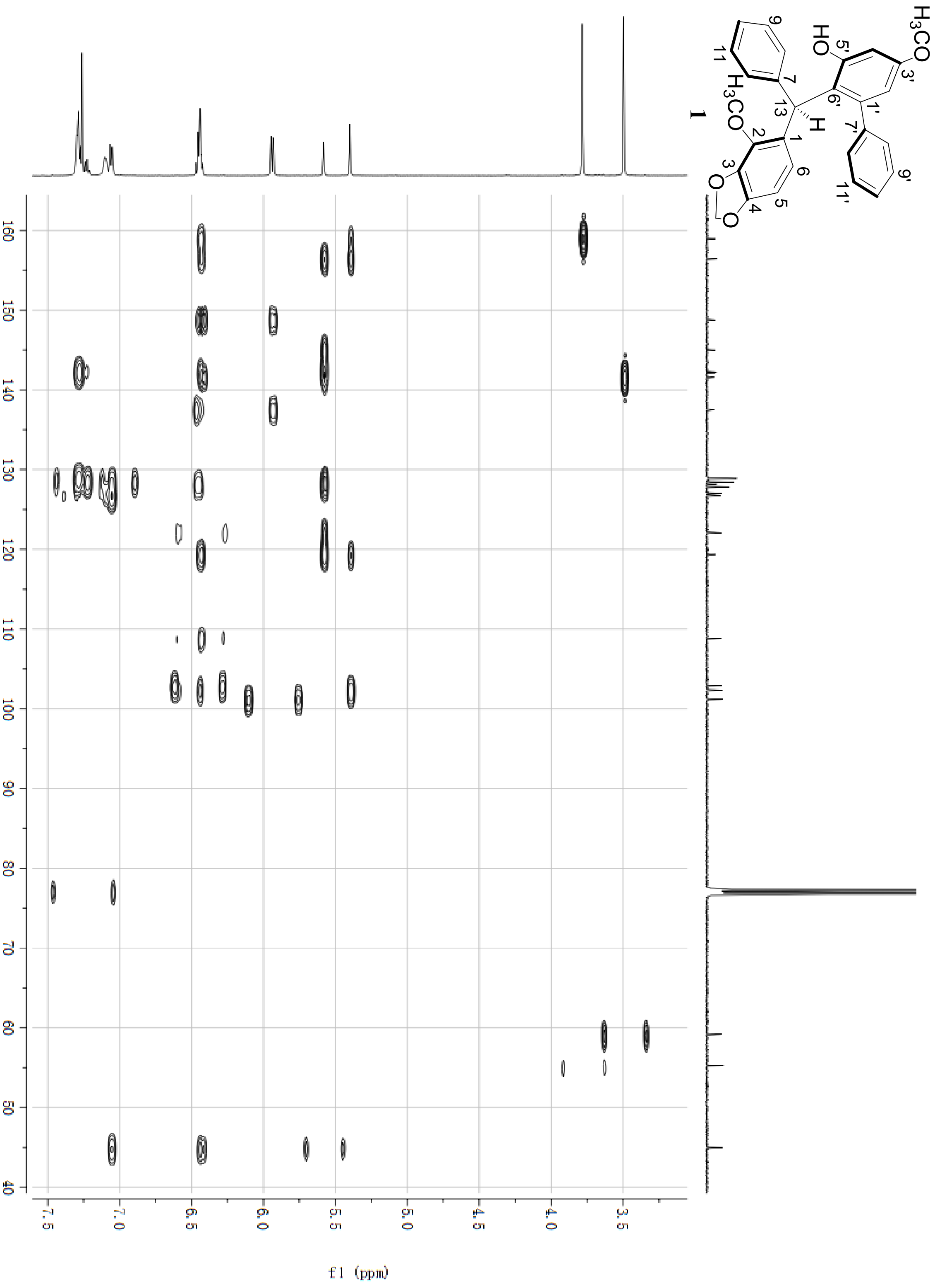

Figure S14. ESI(+)MS spectrum of (−)-securidane A (**1**)

Display Report

Analysis Info

Analysis Name	014-2901.D	Acquisition Date	05/24/15 03:28:58
Method	Copy of DSOPMS2P.M	Operator	Administrator
Sample Name	yjm-CIM-15-1	Instrument	esquire3000plus
Comment	I$⊓		

Acquisition Parameter

Ion Source Type	ESI	Ion Polarity	Positive	Alternating Ion Polarity	off
Mass Range Mode	Std/Normal	Scan Begin	100 m/z	Scan End	1750 m/z
Capillary Exit	158.5 Volt	Skim 1	40.0 Volt	Trap Drive	85.2
Accumulation Time	15000 鍟	Averages	3 Spectra	Auto MS/MS	on

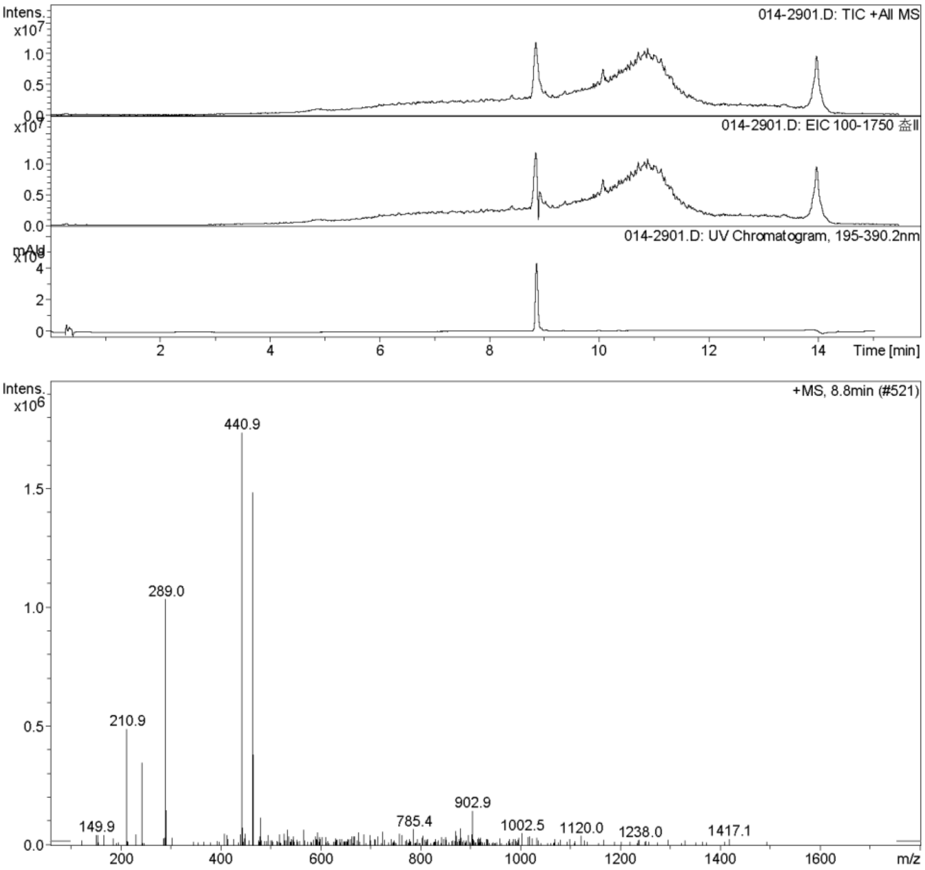

Bruker Daltonics DataAnalysis 3.1 printed: 05/28/15 14:27:07 Page 1 of 1

Figure S15. ESI(−)MS spectrum of (−)-securidane A (**1**)

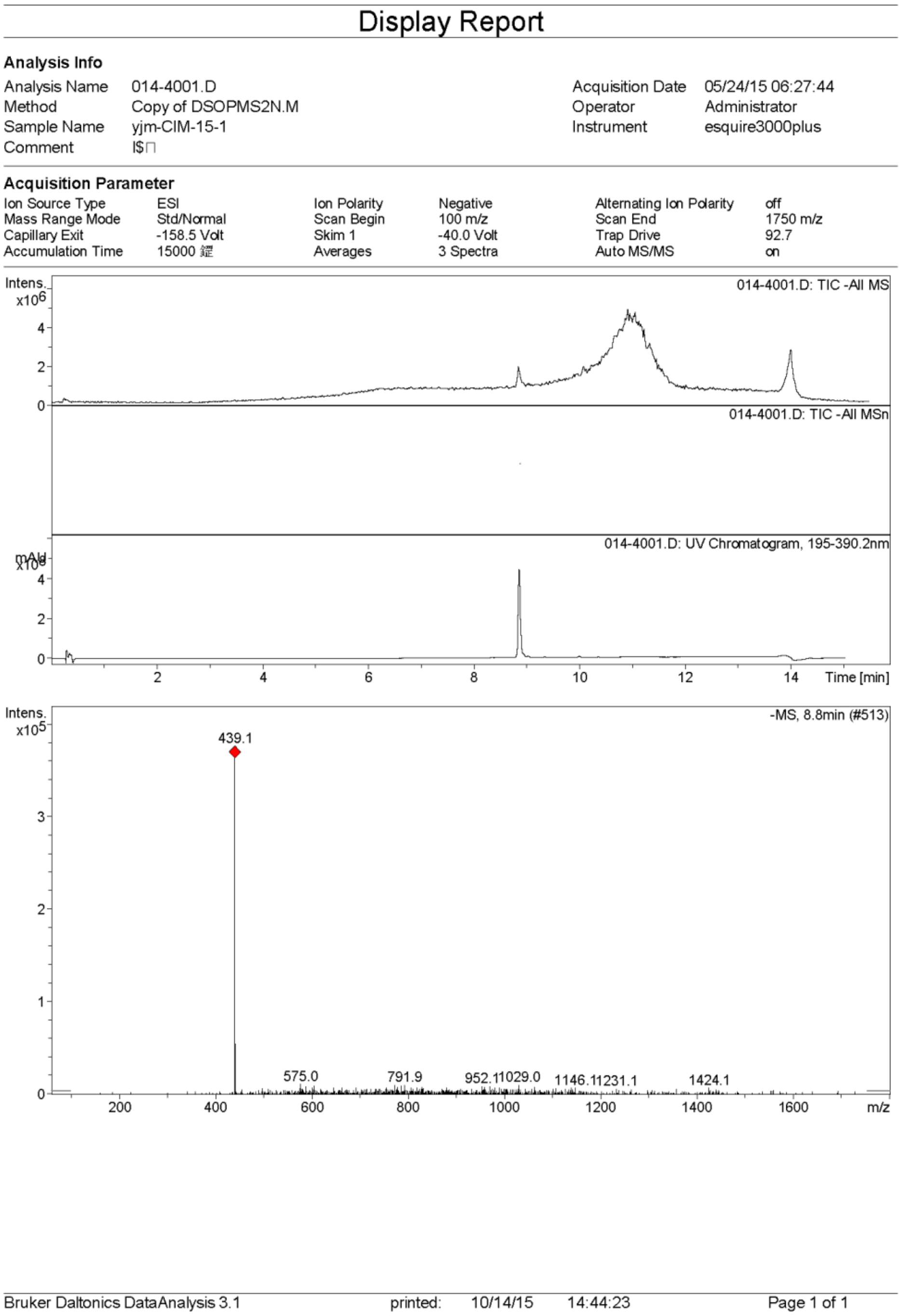

Figure S16. HRESI(+)MS spectrum of (–)-securidane A (**1**)

Elemental Composition Report **Page 1**

Single Mass Analysis
Tolerance = 5.0 PPM / DBE: min = -1.5, max = 50.0
Element prediction: Off
Number of isotope peaks used for i-FIT = 3

Monoisotopic Mass, Even Electron Ions
177 formula(e) evaluated with 1 results within limits (up to 50 closest results for each mass)
Elements Used:
C: 6-60 H: 2-110 O: 0-30 Na: 0-1

CIM-16 LCT PXE KE324 24-Oct-2012 13:55:52

CIM-16_20121024 9 (0.195) AM2 (Ar,10000.0,0.00,1.00); ABS; Cm (8:24) 1: TOF MS ES+ 1.26e+004

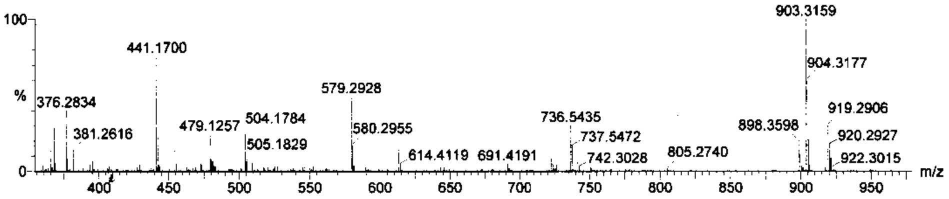

Minimum:				-1.5			
Maximum:		3.0	5.0	50.0			
Mass	Calc. Mass	mDa	PPM	DBE	i-FIT	i-FIT (Norm)	Formula
441.1700	441.1702	-0.2	-0.5	16.5	136.4	0.0	C28 H25 O5

Figure S17. IR spectrum of (−)-securidane A (**1**)

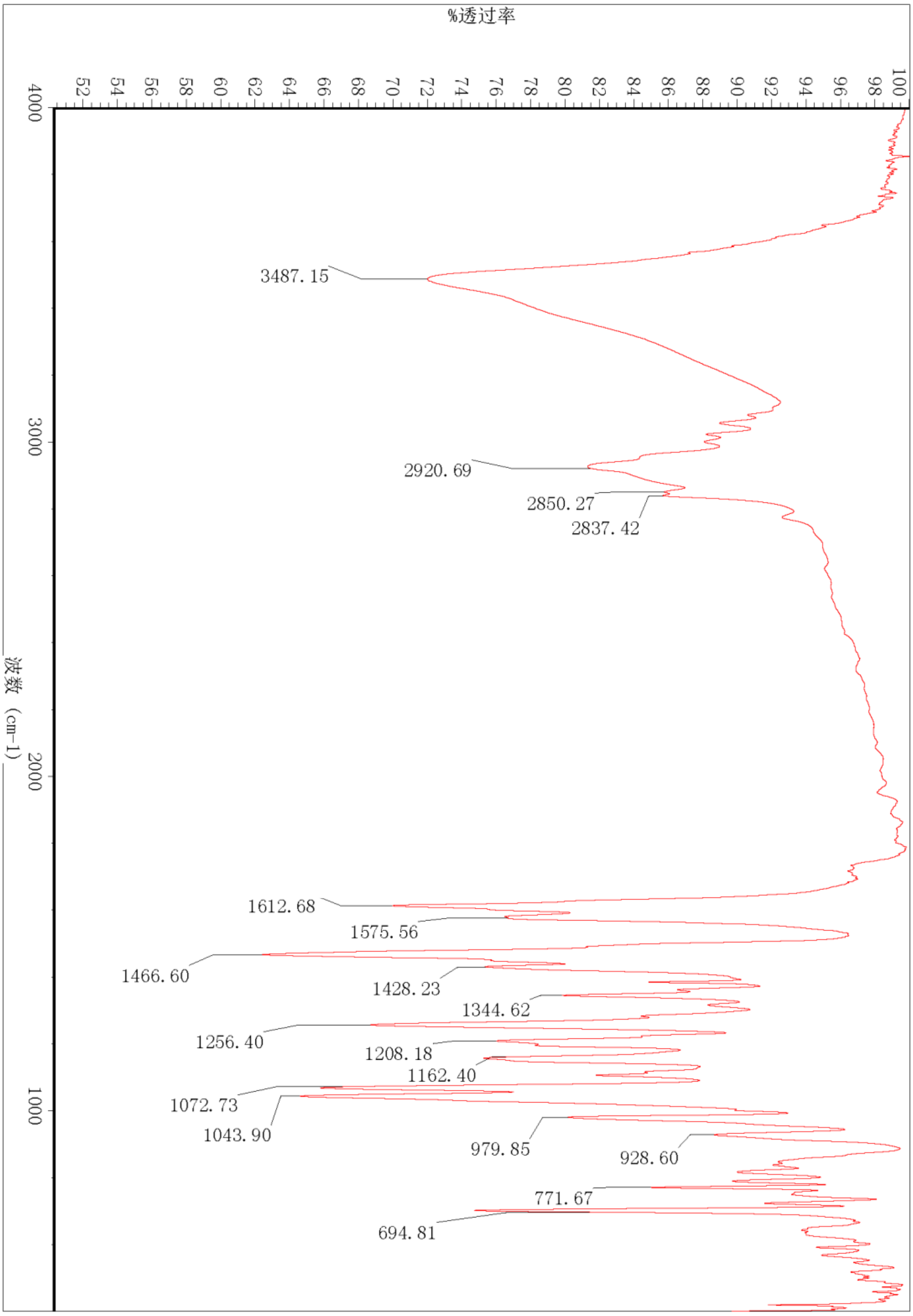

Figure S18. ^{1}H NMR spectrum of (+)-securidane A (**2**) in $CDCl_3$

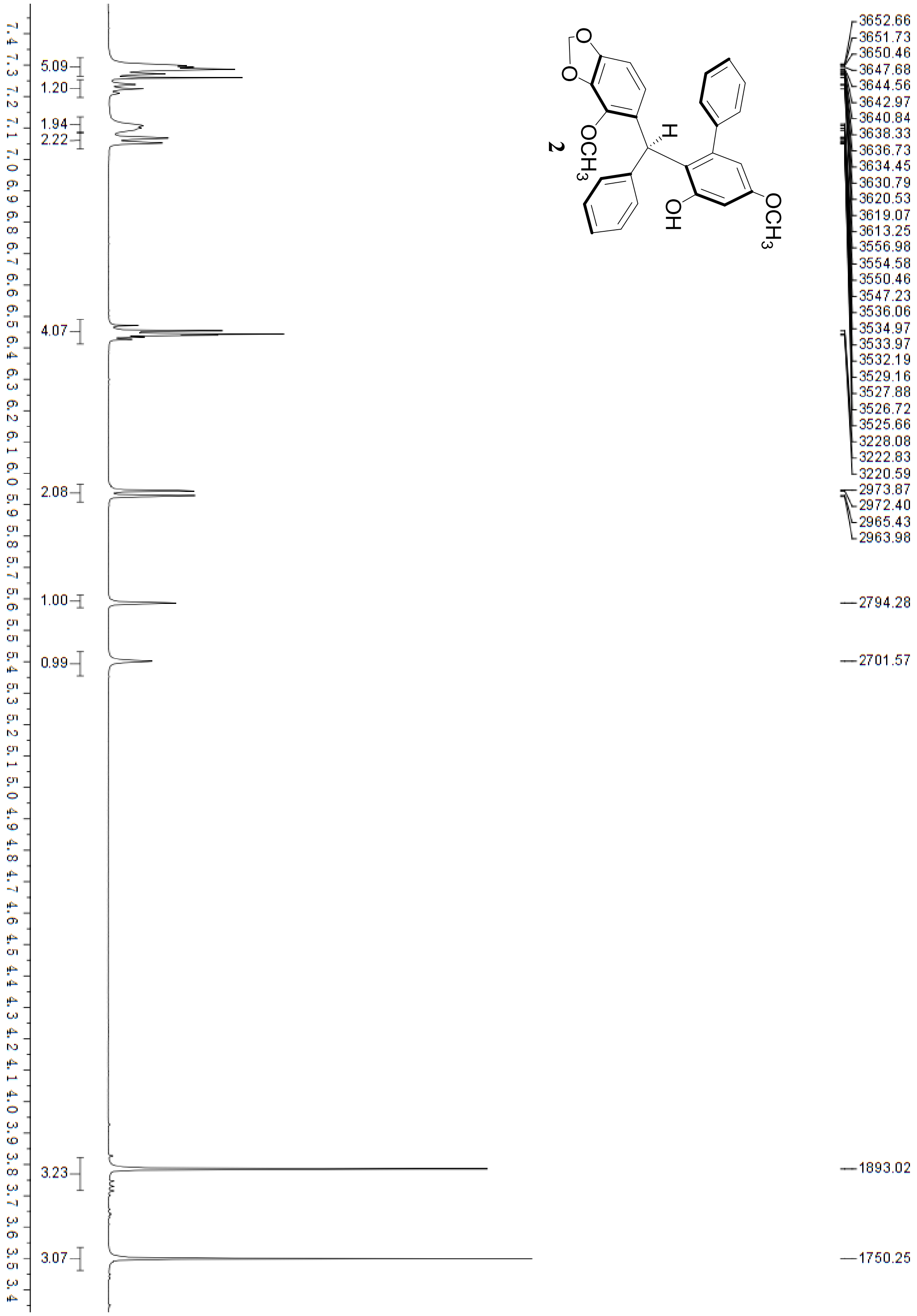

Figure S19. ^{13}C NMR spectrum of (+)-securidane A (**2**) in $CDCl_3$

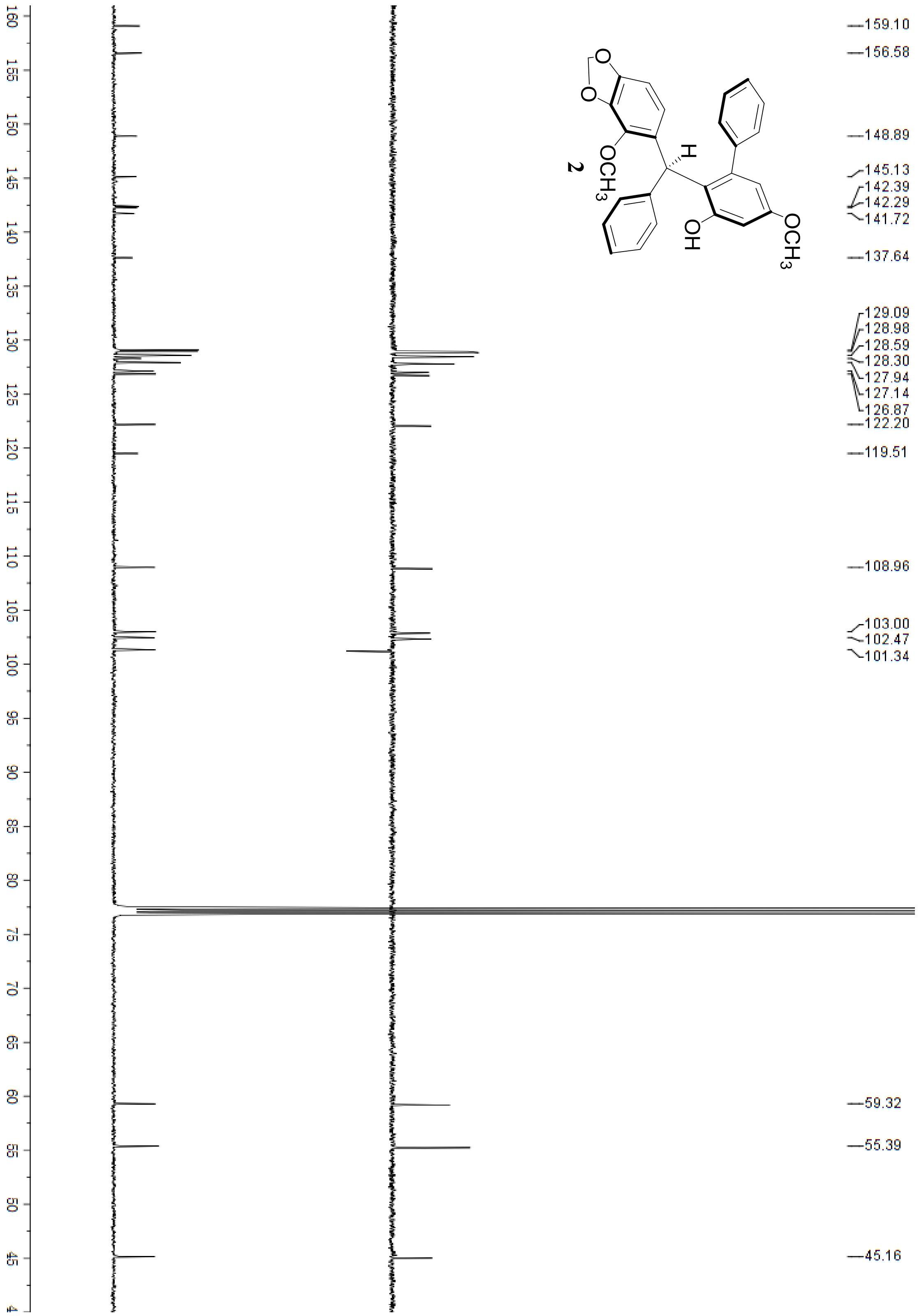

Figure S20. ^{1}H NMR spectrum of (–)-securidane B (**3**) in $CDCl_3$

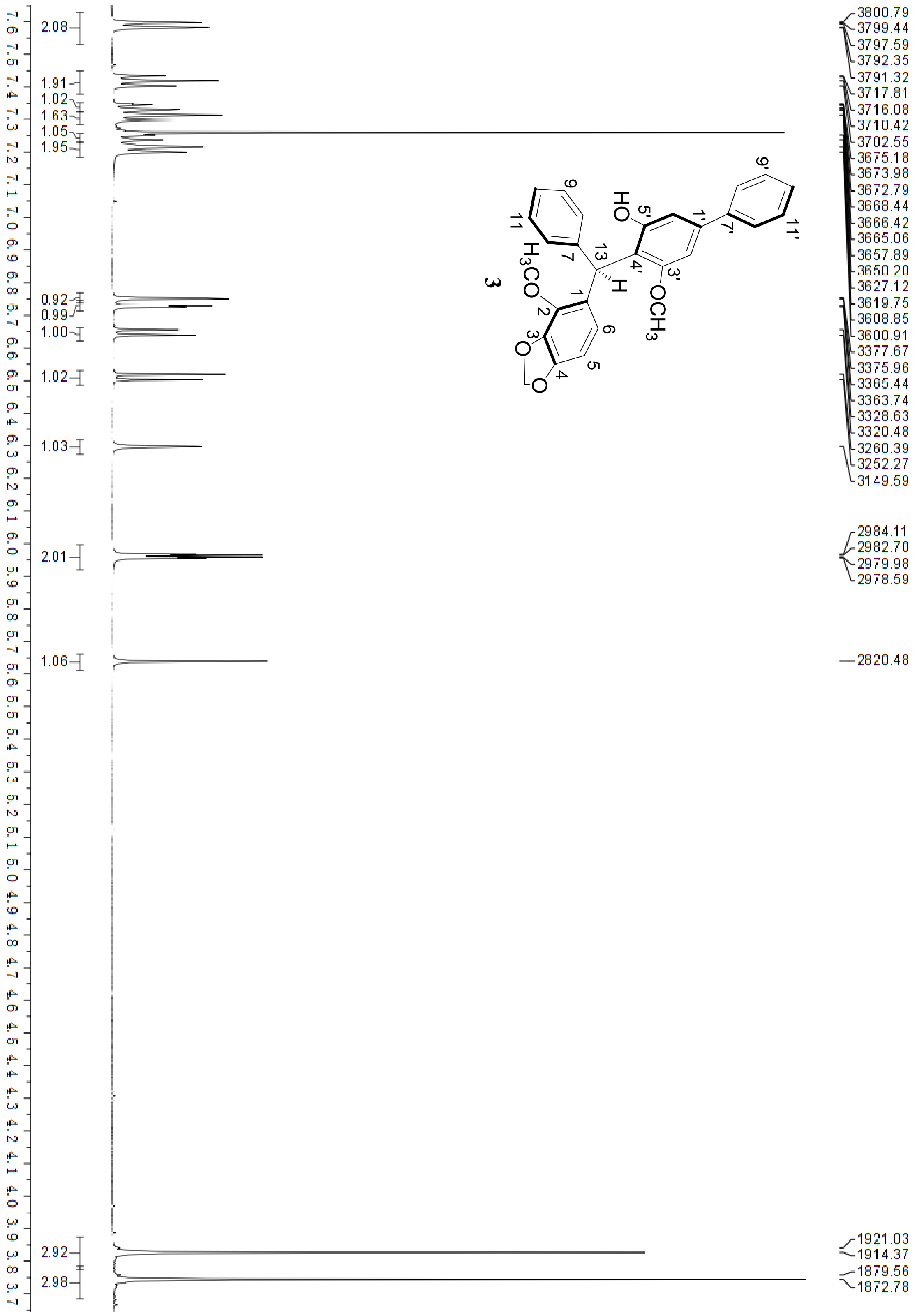

Figure S21. ^{13}C NMR spectrum of (–)-securidane B (**3**) in $CDCl_3$

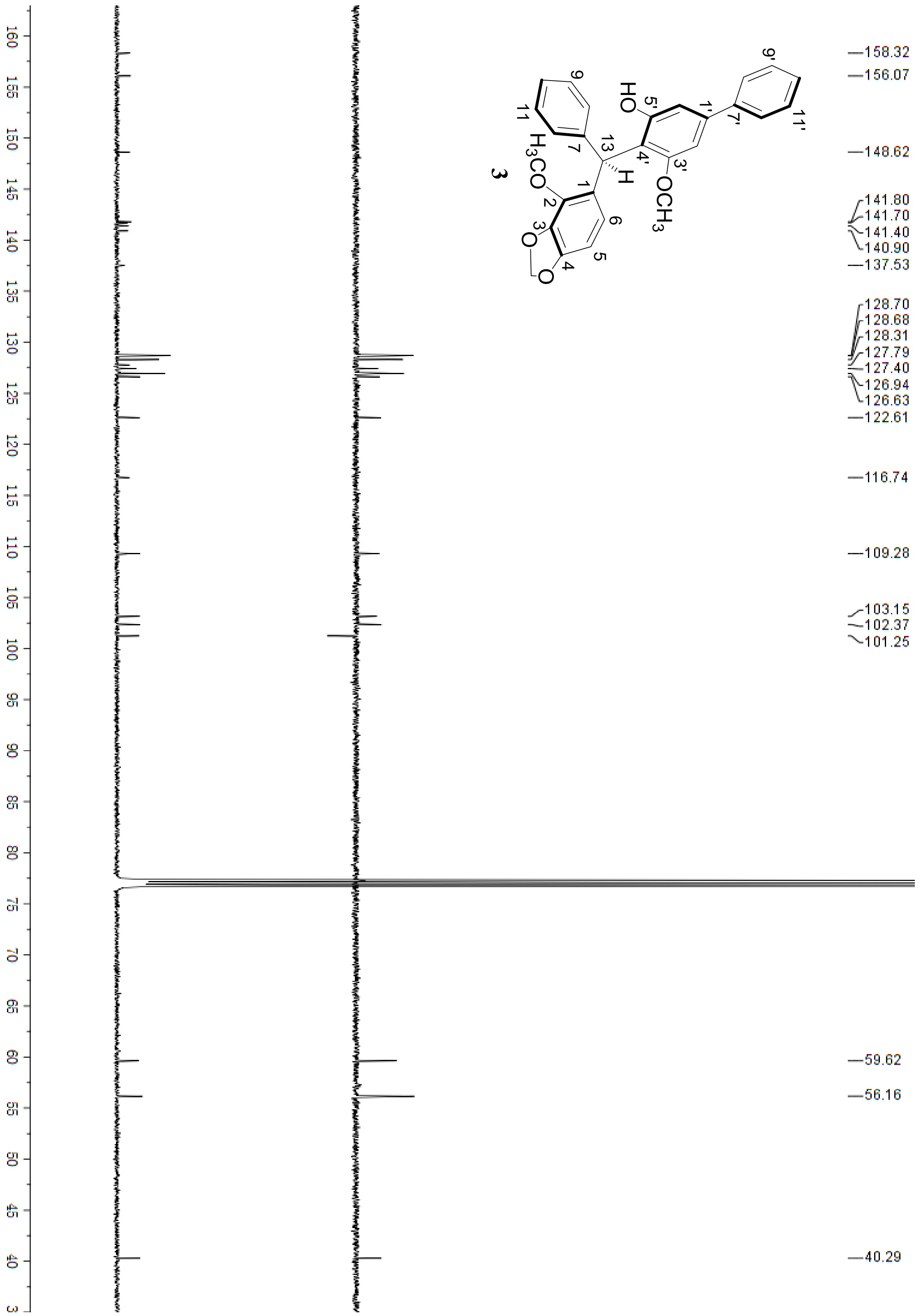

Figure S22. $^{1}H-^{1}H$ COSY spectrum of (–)-securidane B (**3**) in $CDCl_3$

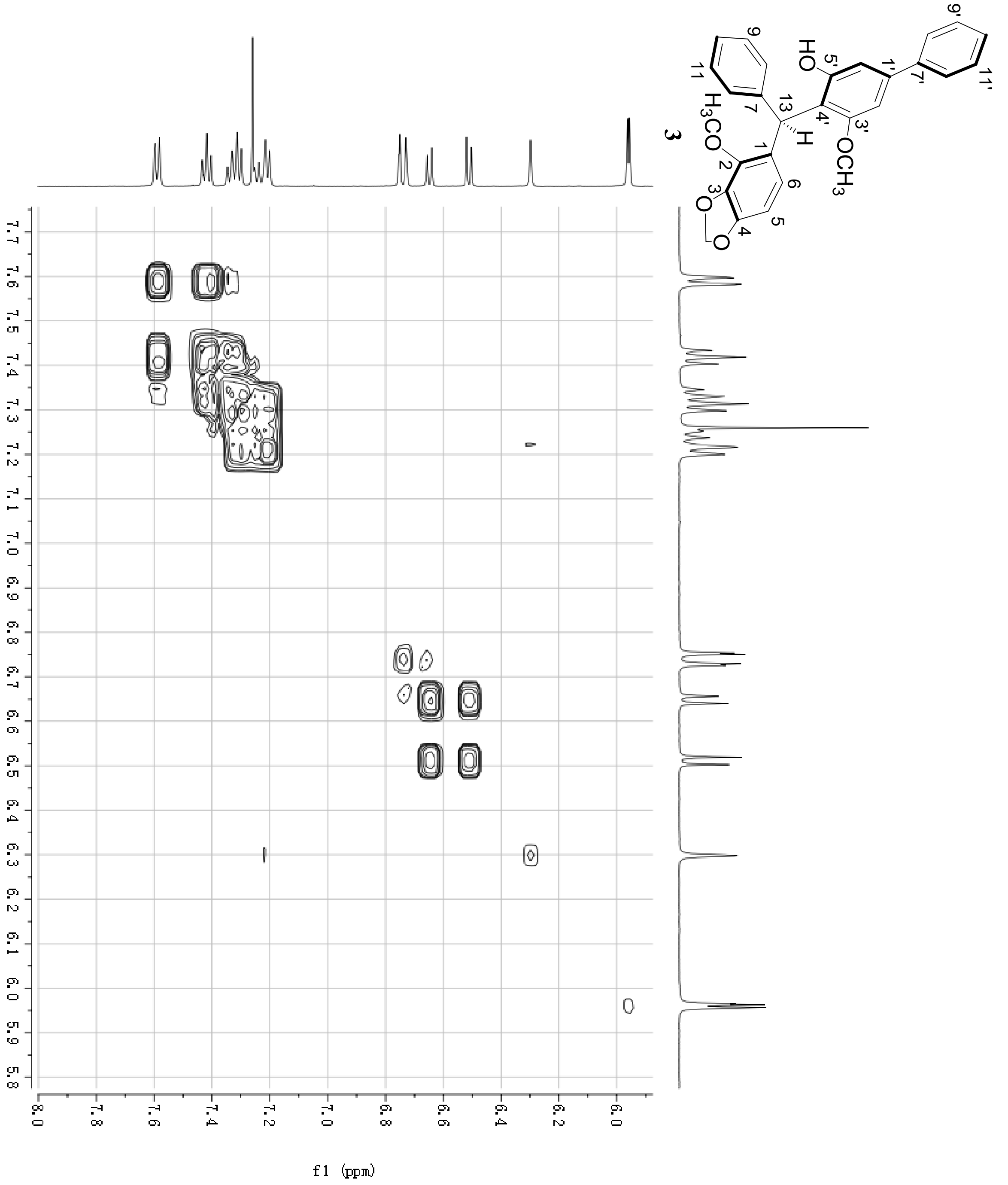

Figure S23. HSQC spectrum of (–)-securidane B (**3**) in $CDCl_3$

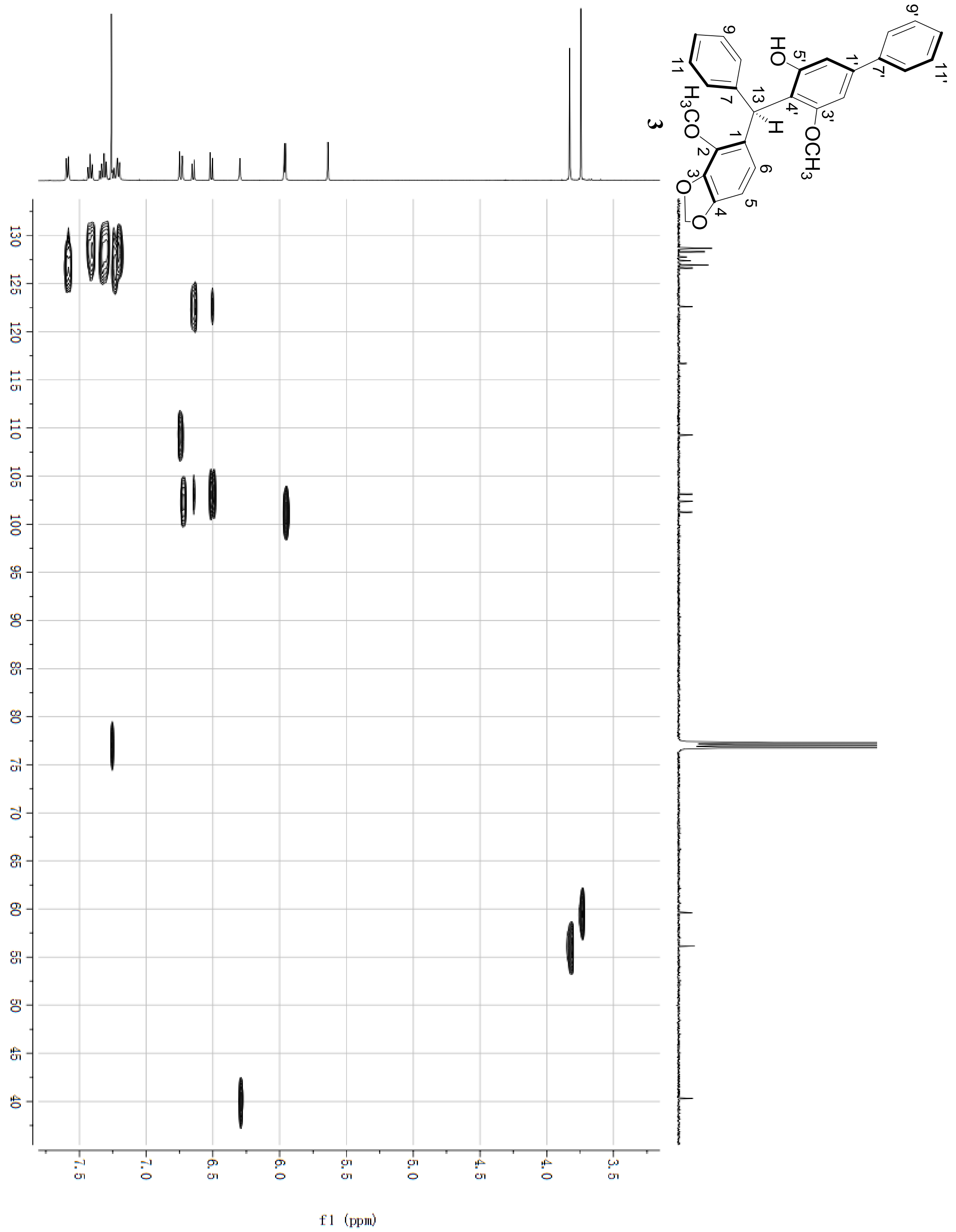

Figure S24. HMBC spectrum of (–)-securidane B (**3**) in $CDCl_3$

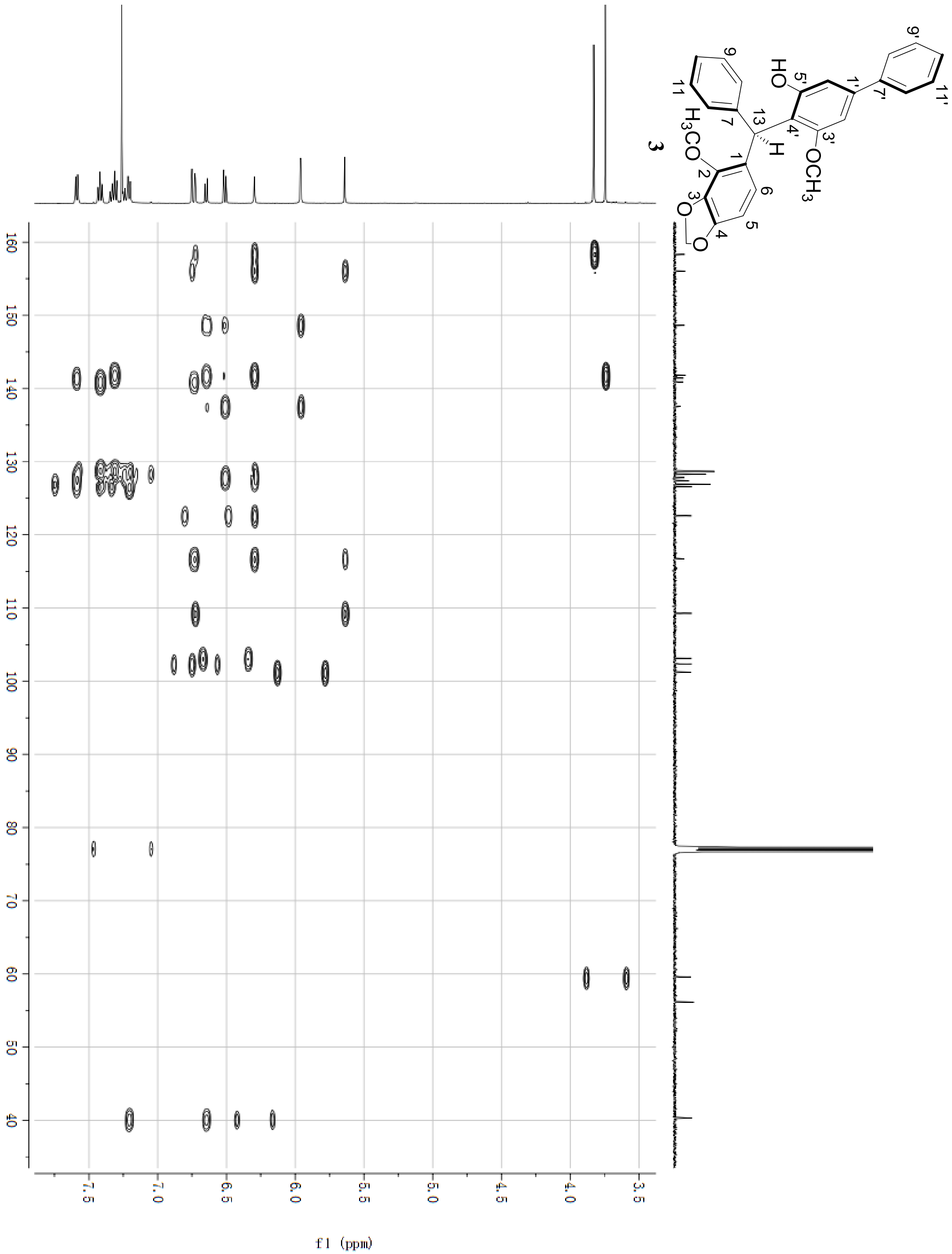

Figure S25. ESI(+)MS spectrum of (–)-securidane B (**3**)

Display Report

Analysis Info

Analysis Name	015-3001.D	Acquisition Date	05/24/15 03:45:12
Method	Copy of DSOPMS2P.M	Operator	Administrator
Sample Name	yjm-CIM-15-3	Instrument	esquire3000plus
Comment	I$⊓		

Acquisition Parameter

Ion Source Type	ESI	Ion Polarity	Positive	Alternating Ion Polarity	off
Mass Range Mode	Std/Normal	Scan Begin	100 m/z	Scan End	1750 m/z
Capillary Exit	158.5 Volt	Skim 1	40.0 Volt	Trap Drive	85.2
Accumulation Time	15000 鋌	Averages	3 Spectra	Auto MS/MS	on

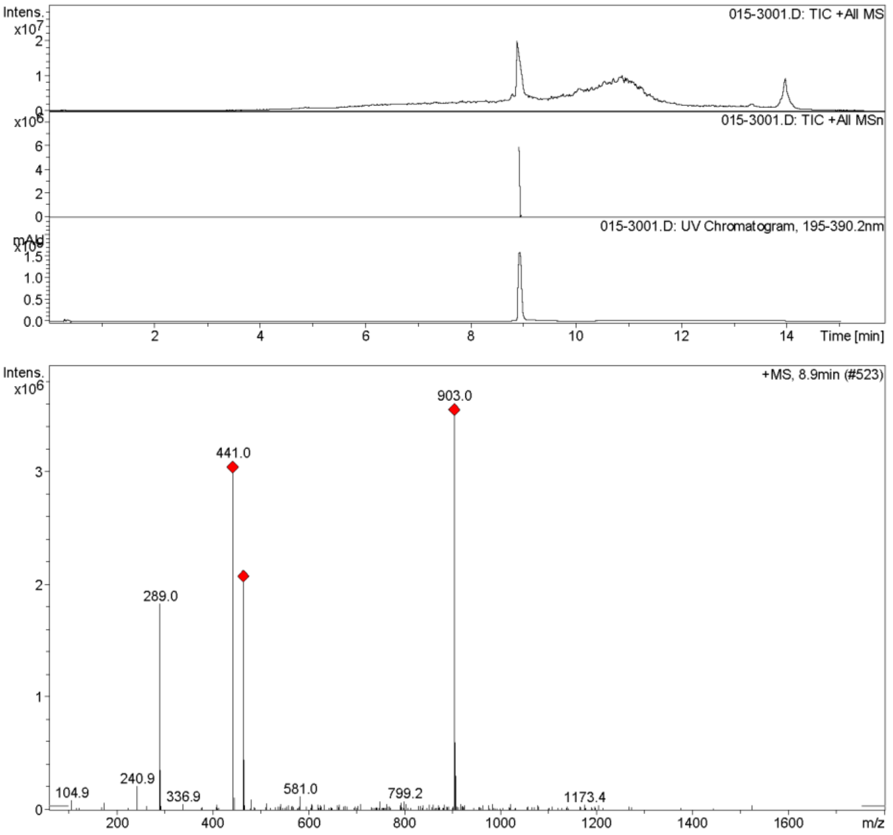

Bruker Daltonics DataAnalysis 3.1 printed: 05/28/15 14:32:06 Page 1 of 1

Figure S26. ESI(−)MS spectrum of (−)-securidane B (**3**)

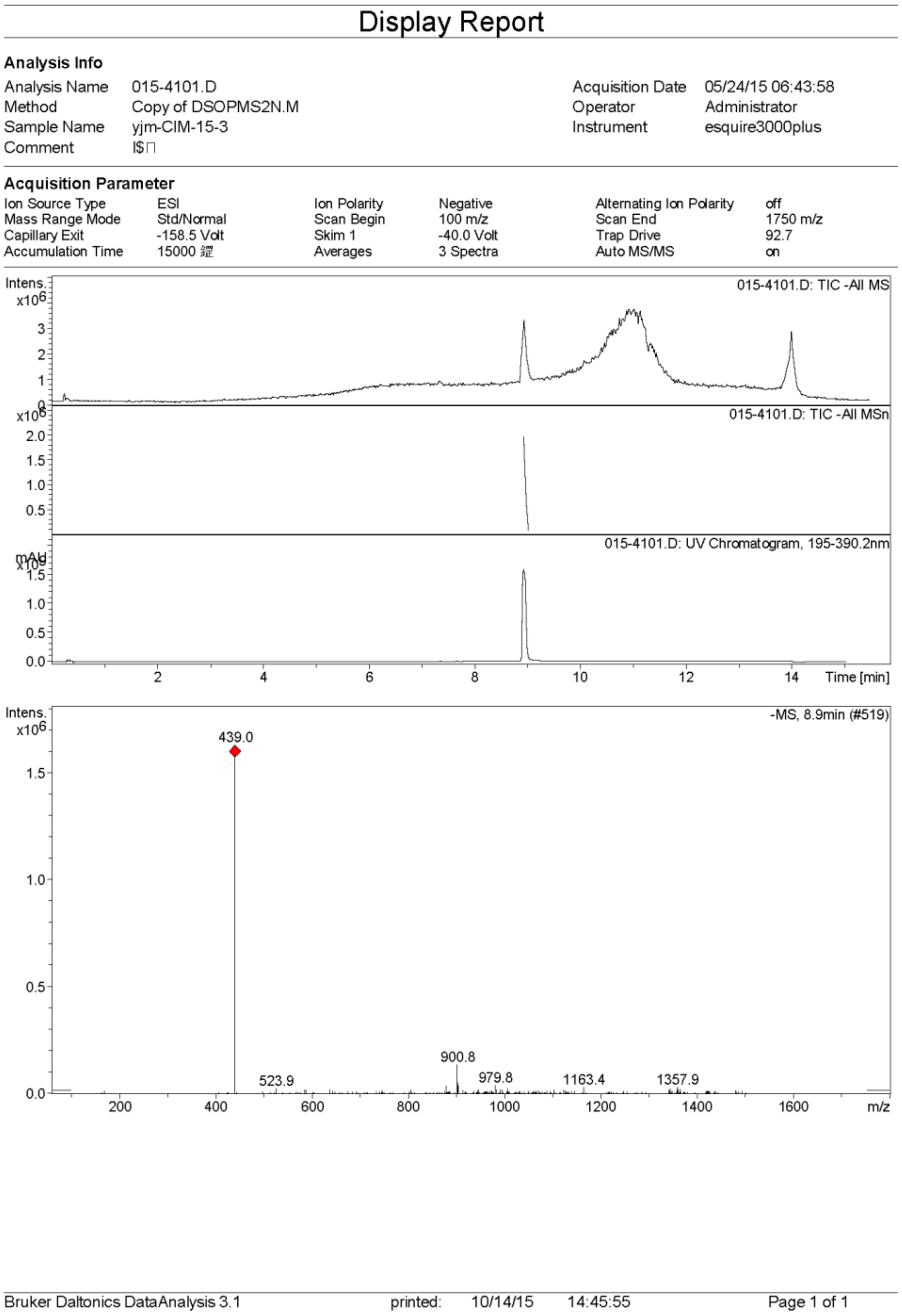

Figure S27. HRESI(+)MS spectrum of (–)-securidane B (**3**)

Elemental Composition Report **Page 1**

Single Mass Analysis
Tolerance = 3.0 PPM / DBE: min = -1.5, max = 50.0
Element prediction: Off
Number of isotope peaks used for i-FIT = 3

Monoisotopic Mass, Even Electron Ions
177 formula(e) evaluated with 1 results within limits (up to 50 closest results for each mass)
Elements Used:
C: 6-60 H: 2-110 O: 0-30 Na: 0-1

CIM-15 LCT PXE KE324 24-Oct-2012
10:56:20
CIM-15_20121024 33 (0.725) AM2 (Ar,10000.0,0.00,1.00); ABS; Cm (20:40) 1: TOF MS ES+
2.46e+004

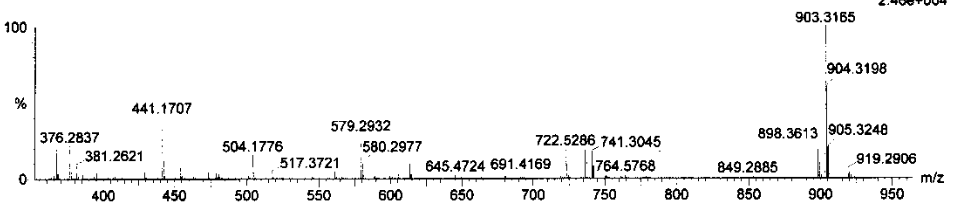

Minimum:				-1.5			
Maximum:		3.0	3.0	50.0			
Mass	Calc. Mass	mDa	PPM	DBE	i-FIT	i-FIT (Norm)	Formula
441.1707	441.1702	0.5	1.1	16.5	110.9	0.0	C28 H25 O5

Figure S28. IR spectrum of (−)-securidane B (**3**)

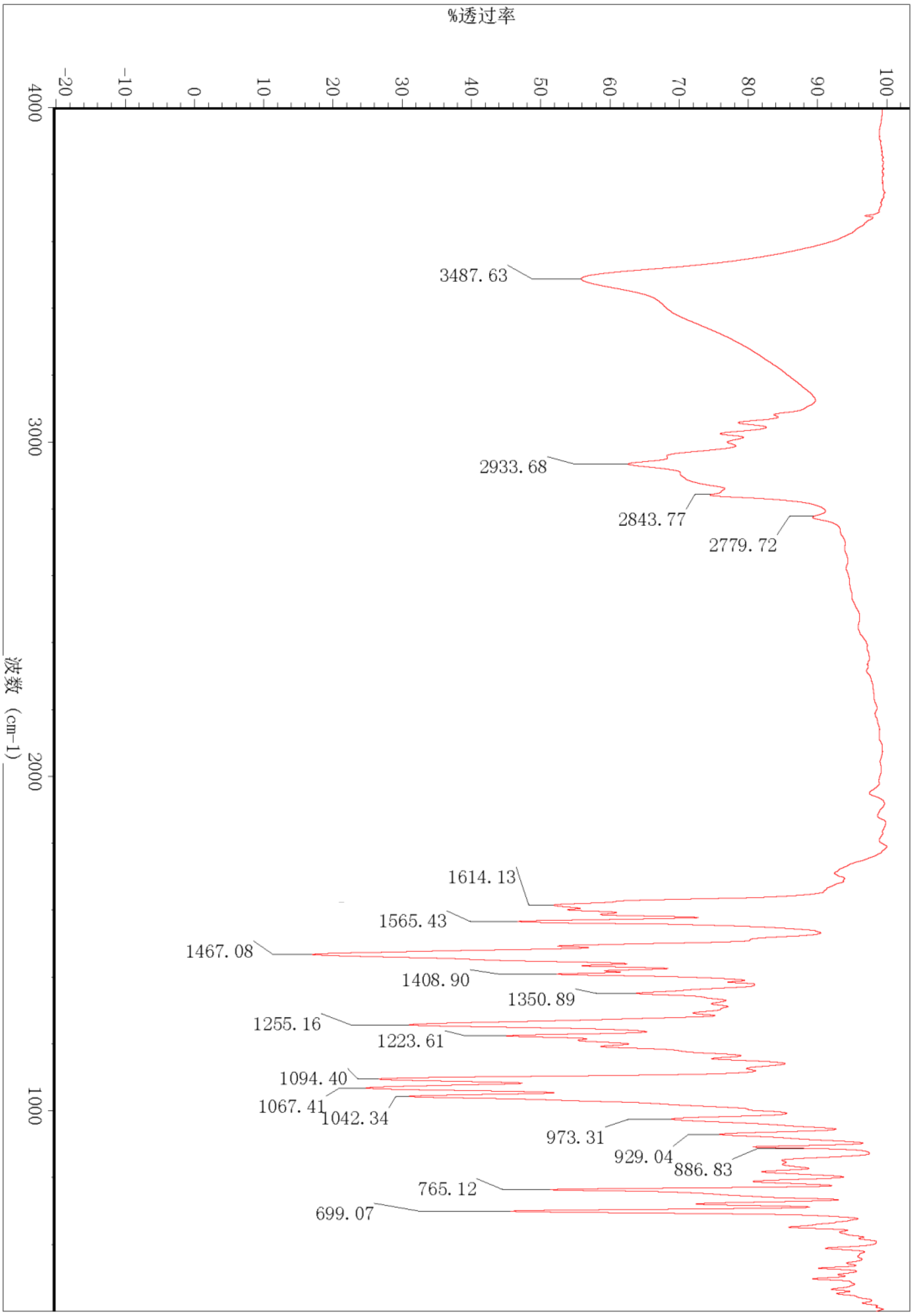

Figure S29. ^{1}H NMR spectrum of (+)-securidane B (**4**) in $CDCl_3$

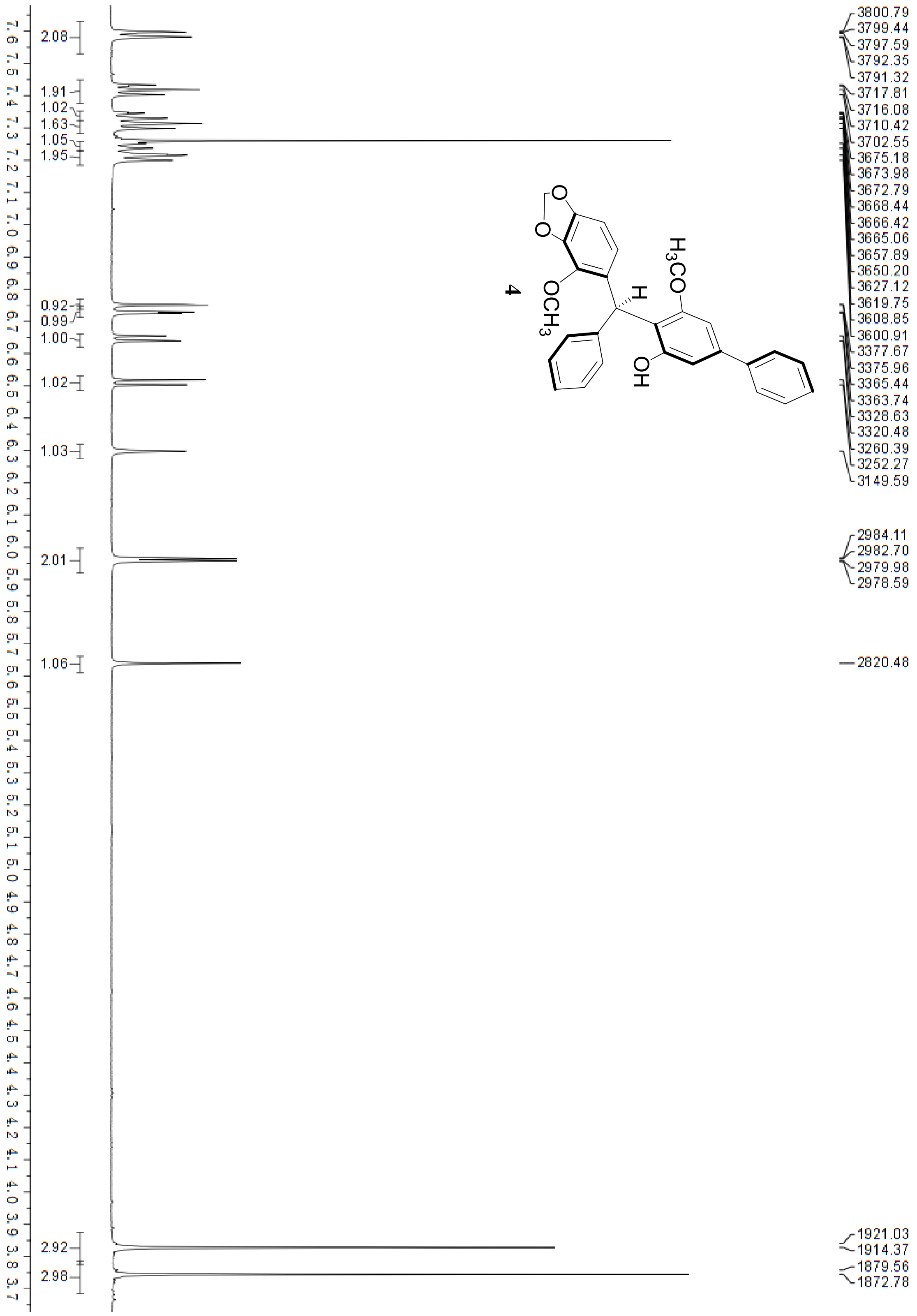

Figure S30. ^{13}C NMR spectrum of (+)-securidane B (**4**) in $CDCl_3$

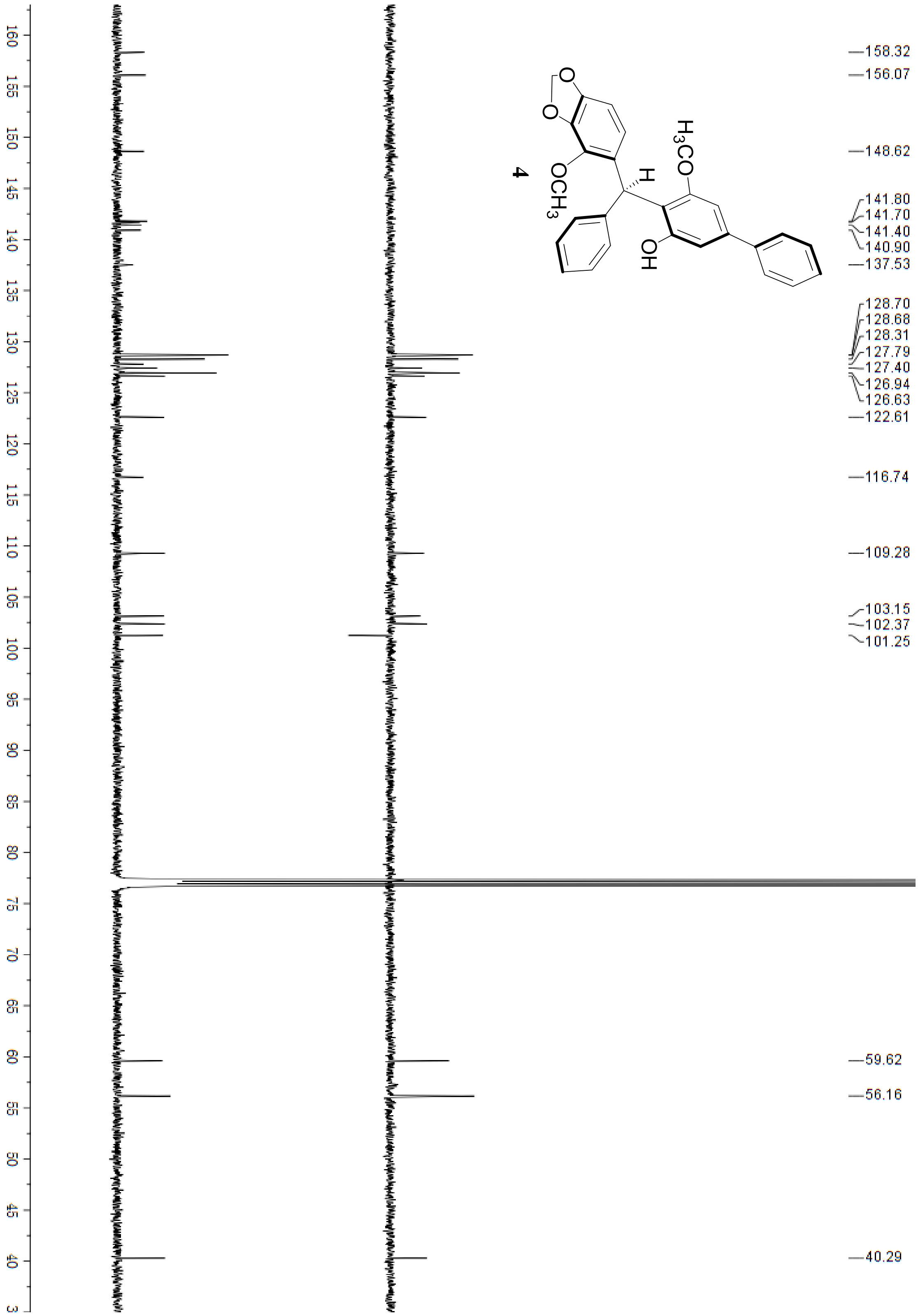

Figure S31. ^{1}H NMR spectrum of compound **11** in $CDCl_3$

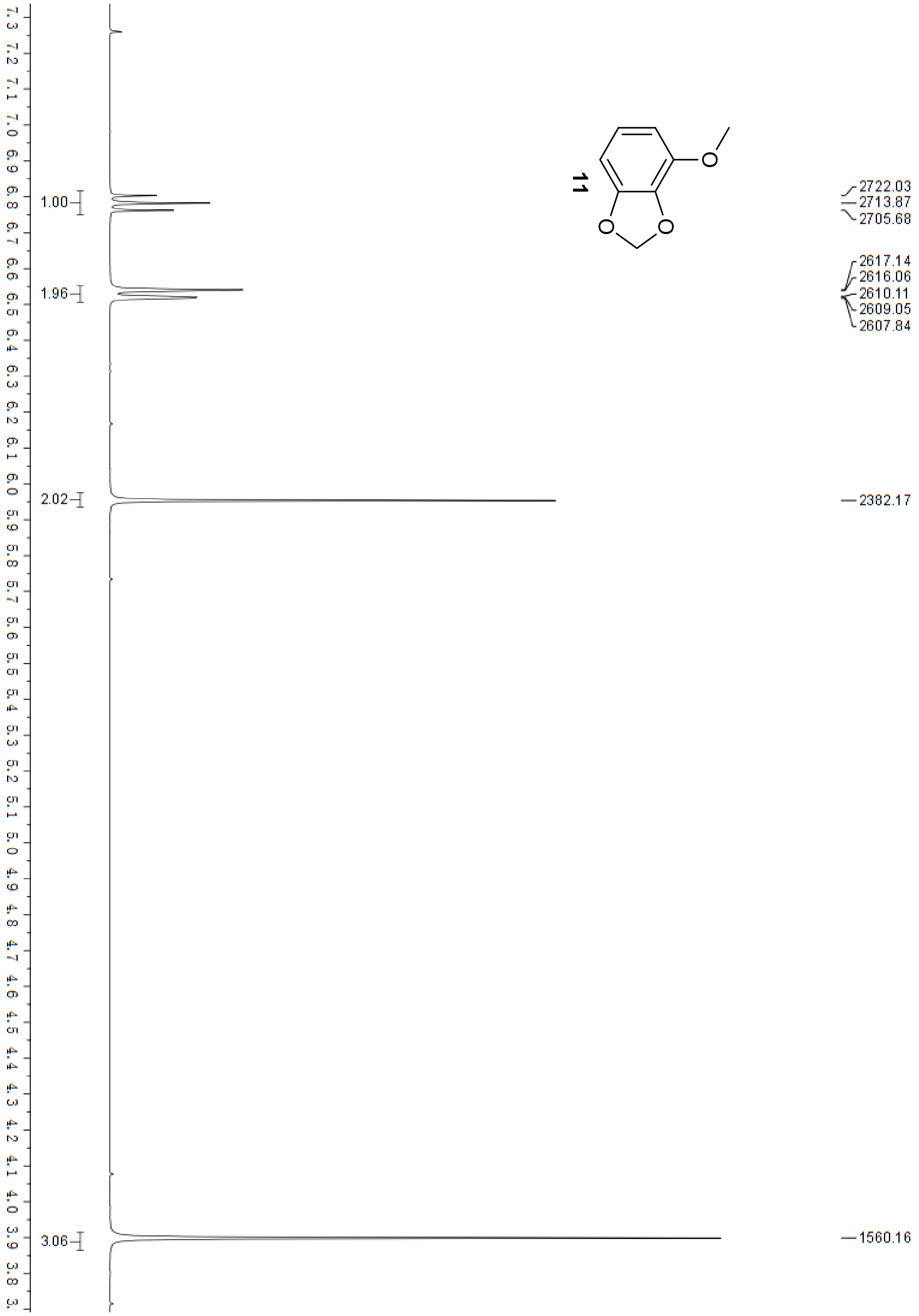

Figure S32. ^{13}C NMR spectrum of compound **11** in $CDCl_3$

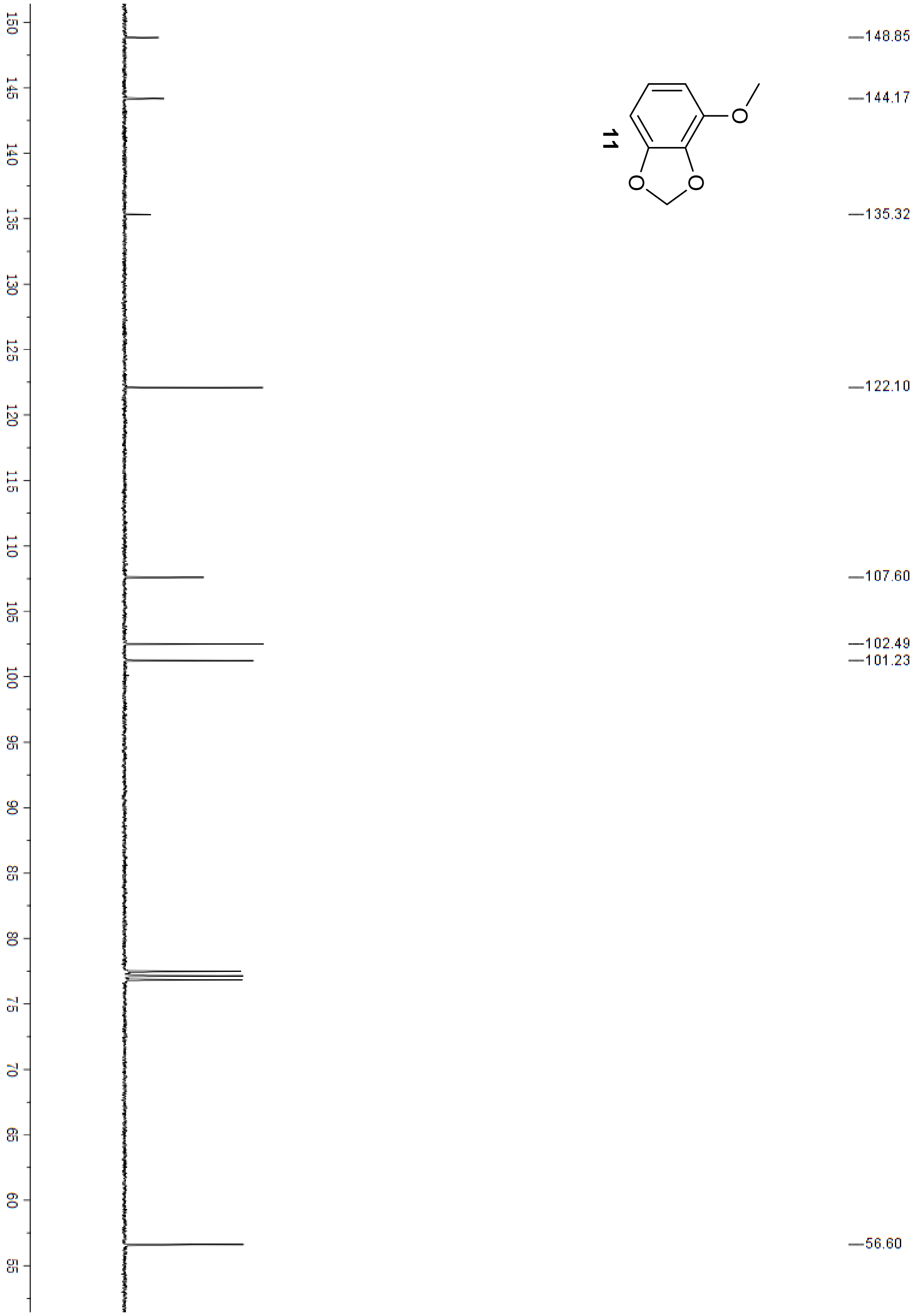

Figure S33. ^{1}H NMR spectrum of compound **10** in $CDCl_3$

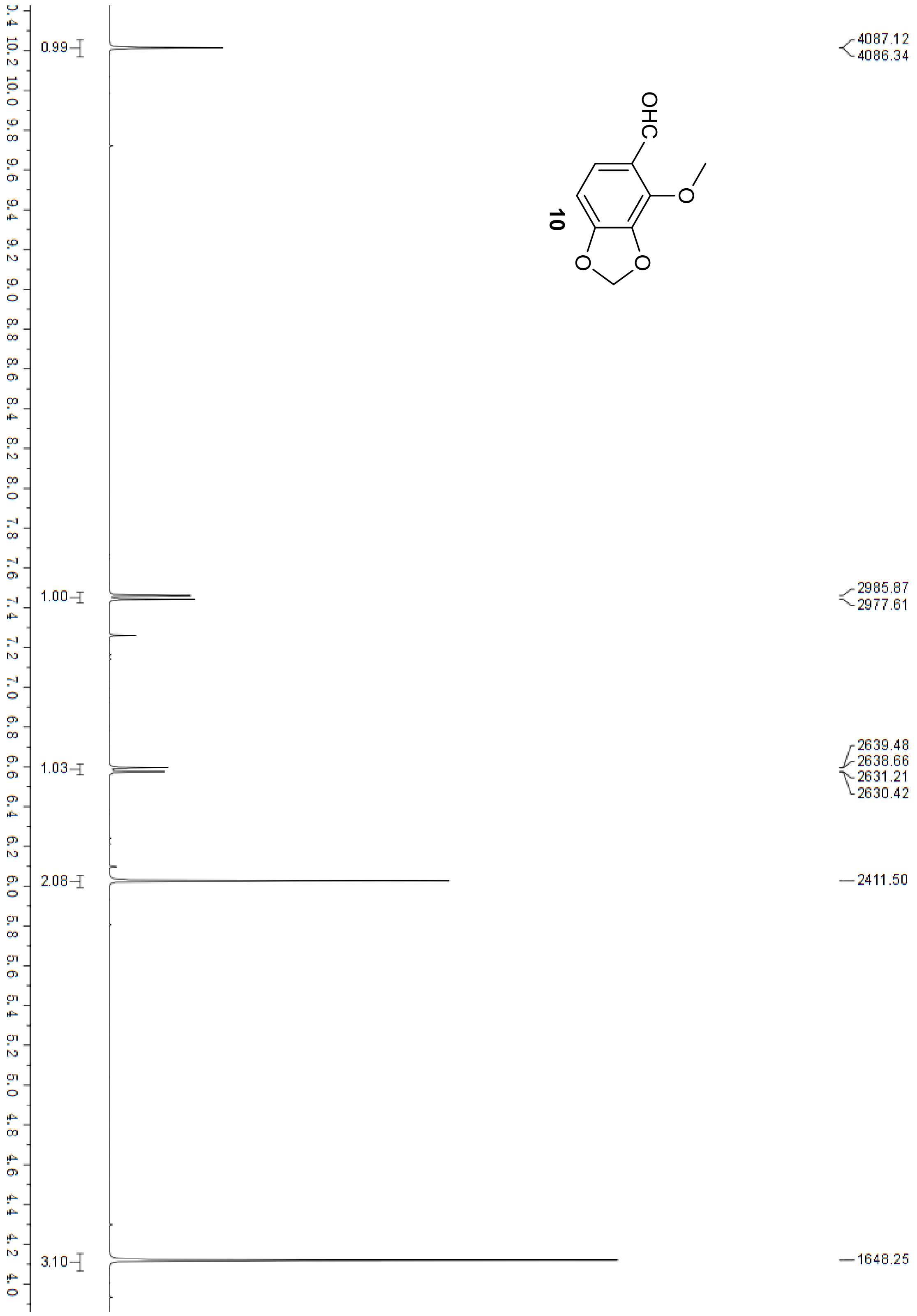

Figure S34. ^{13}C NMR spectrum of compound **10** in $CDCl_3$

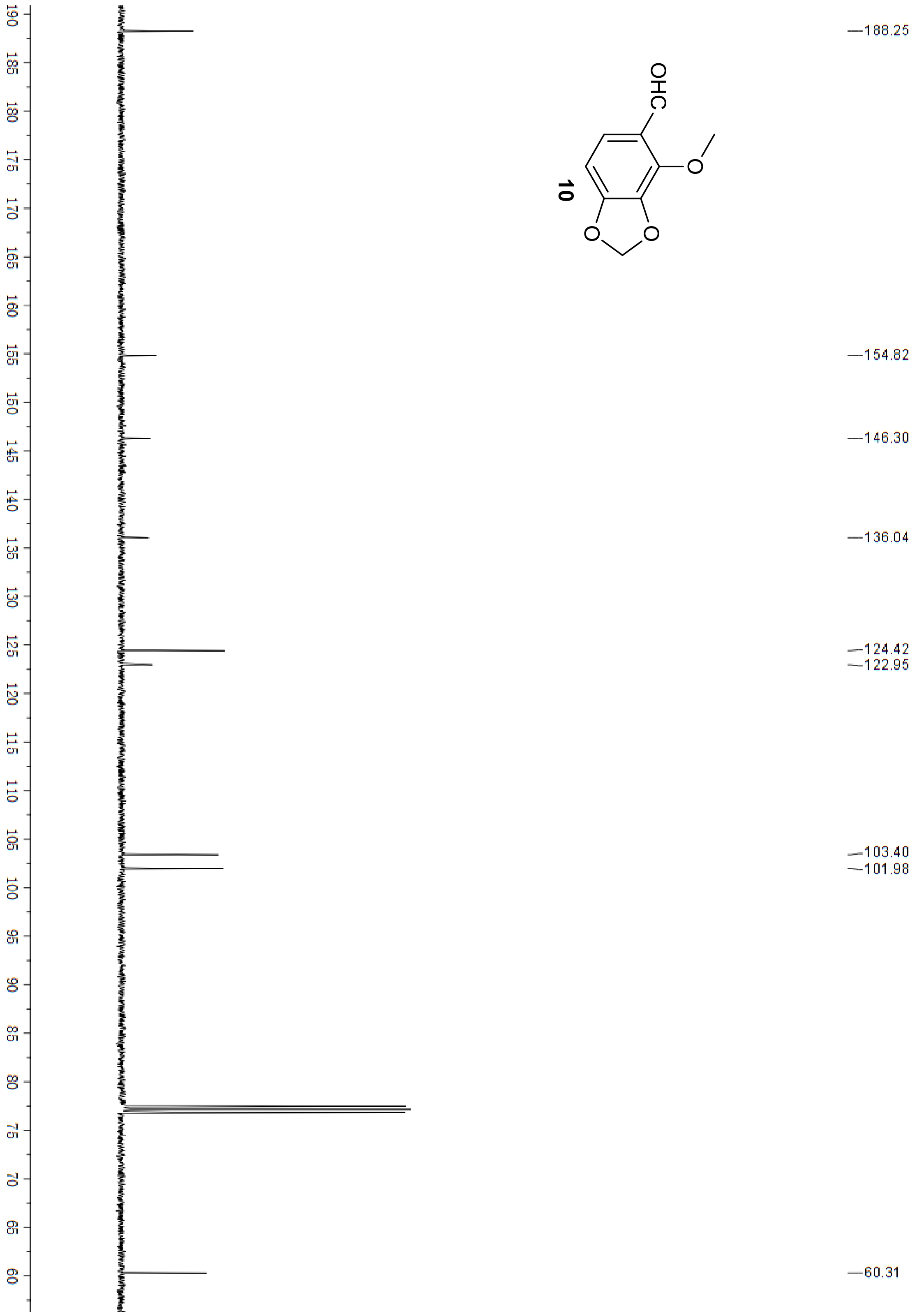

Figure S35. ¹H NMR spectrum of compound **7** in $CDCl_3$

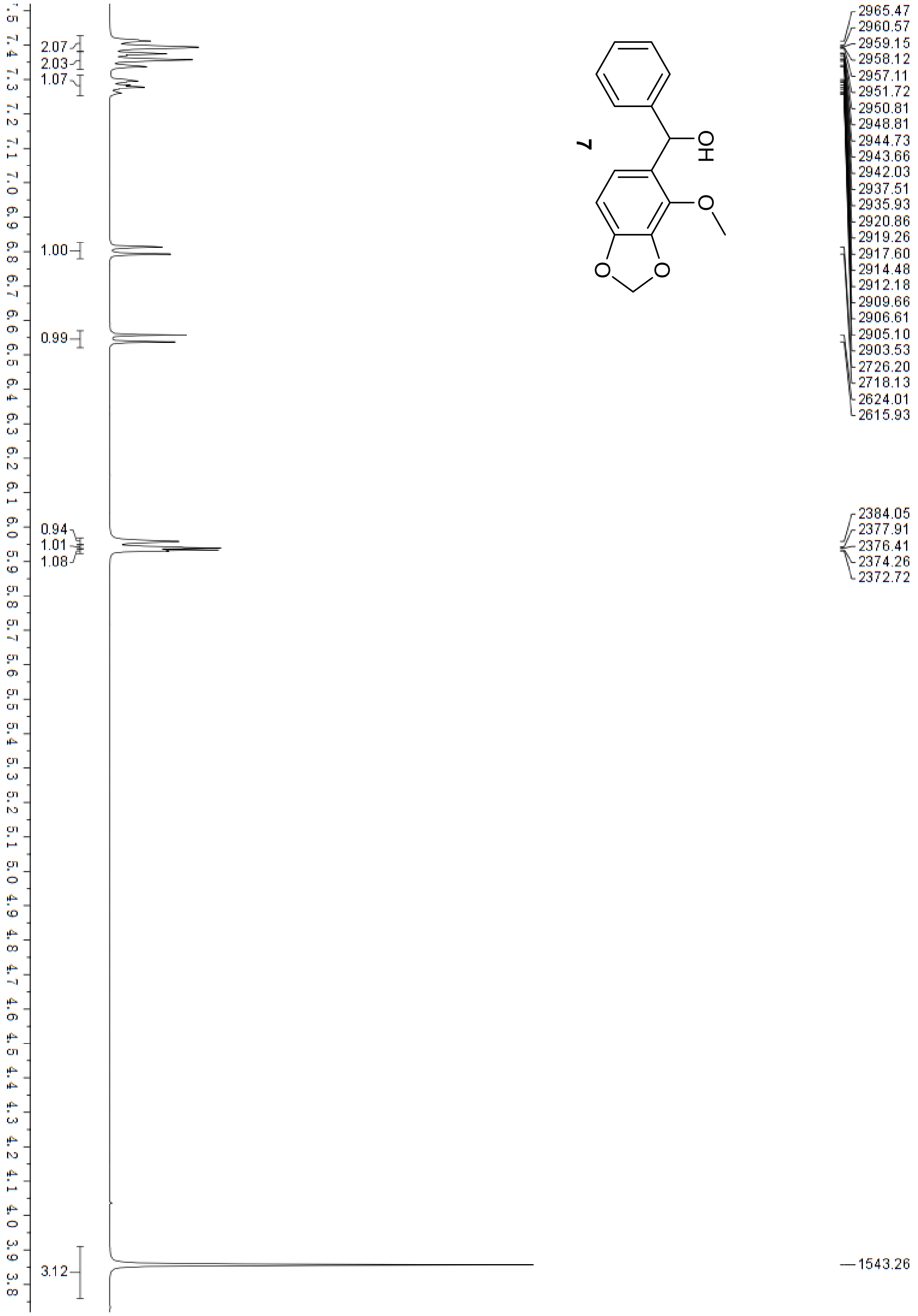

Figure S36. ^{13}C NMR spectrum of compound **7** in $CDCl_3$

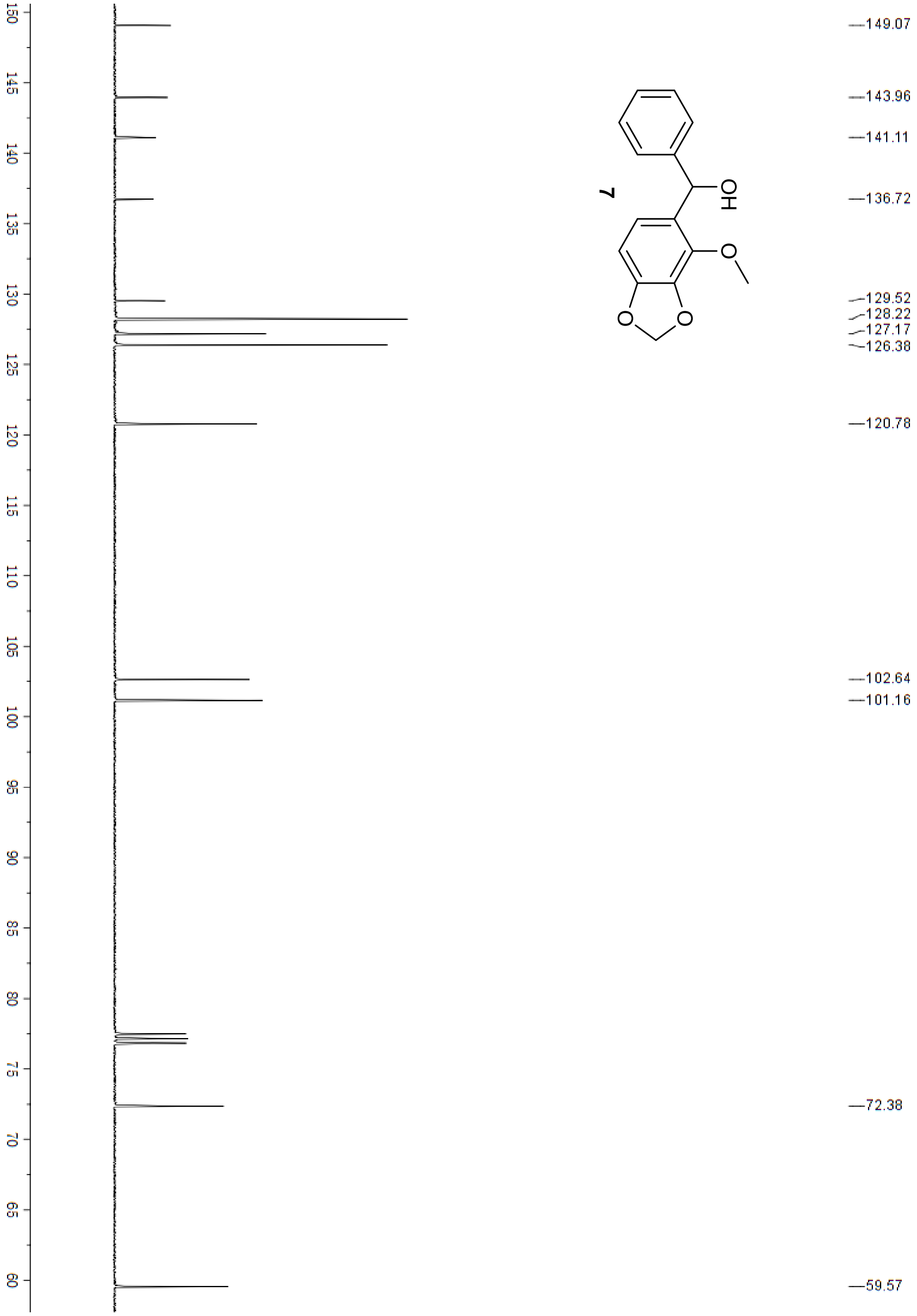

Figure S37. ^{1}H NMR spectrum of compound **6** in $CDCl_3$

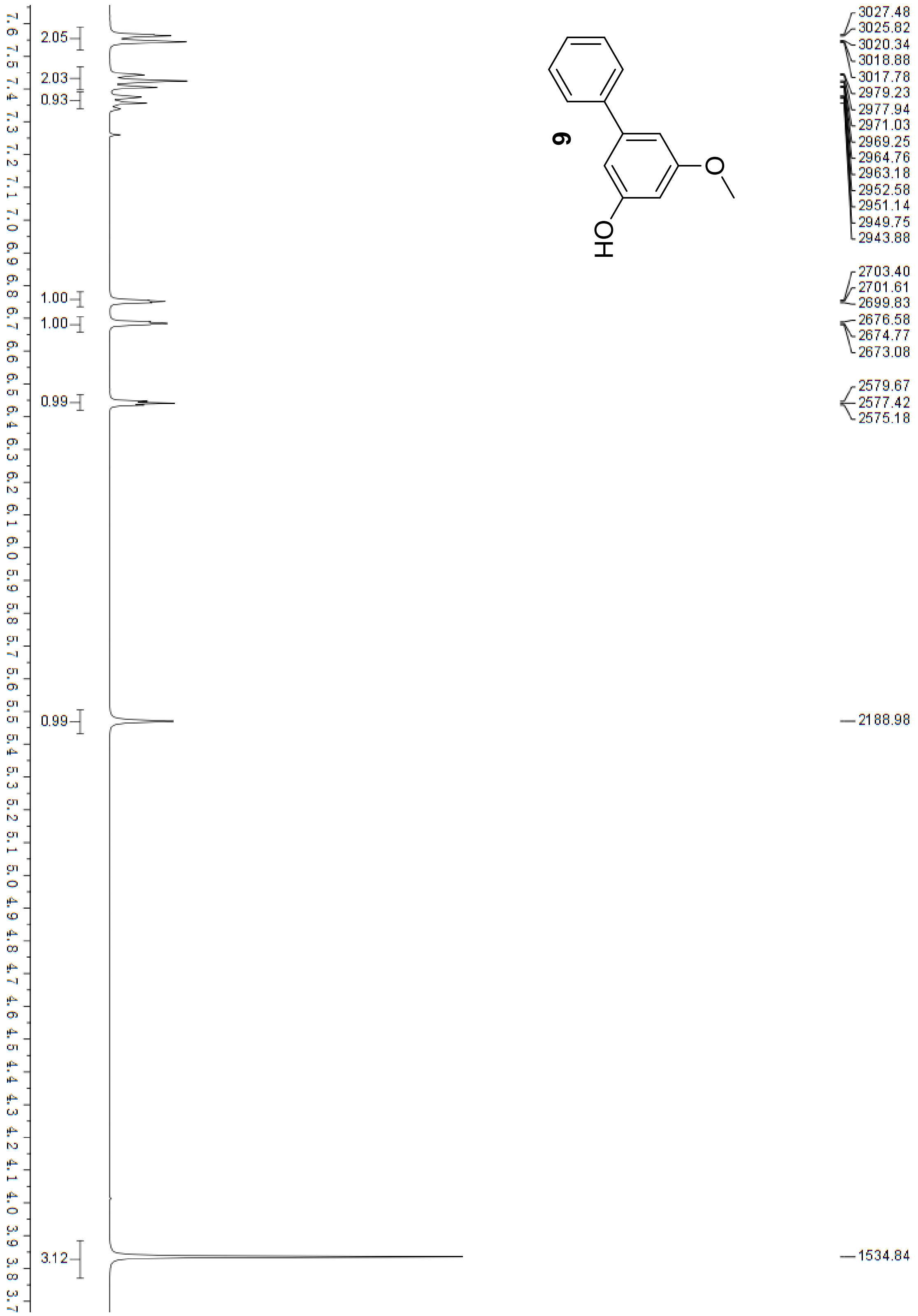

Figure S38. ^{13}C NMR spectrum of compound **6** in $CDCl_3$

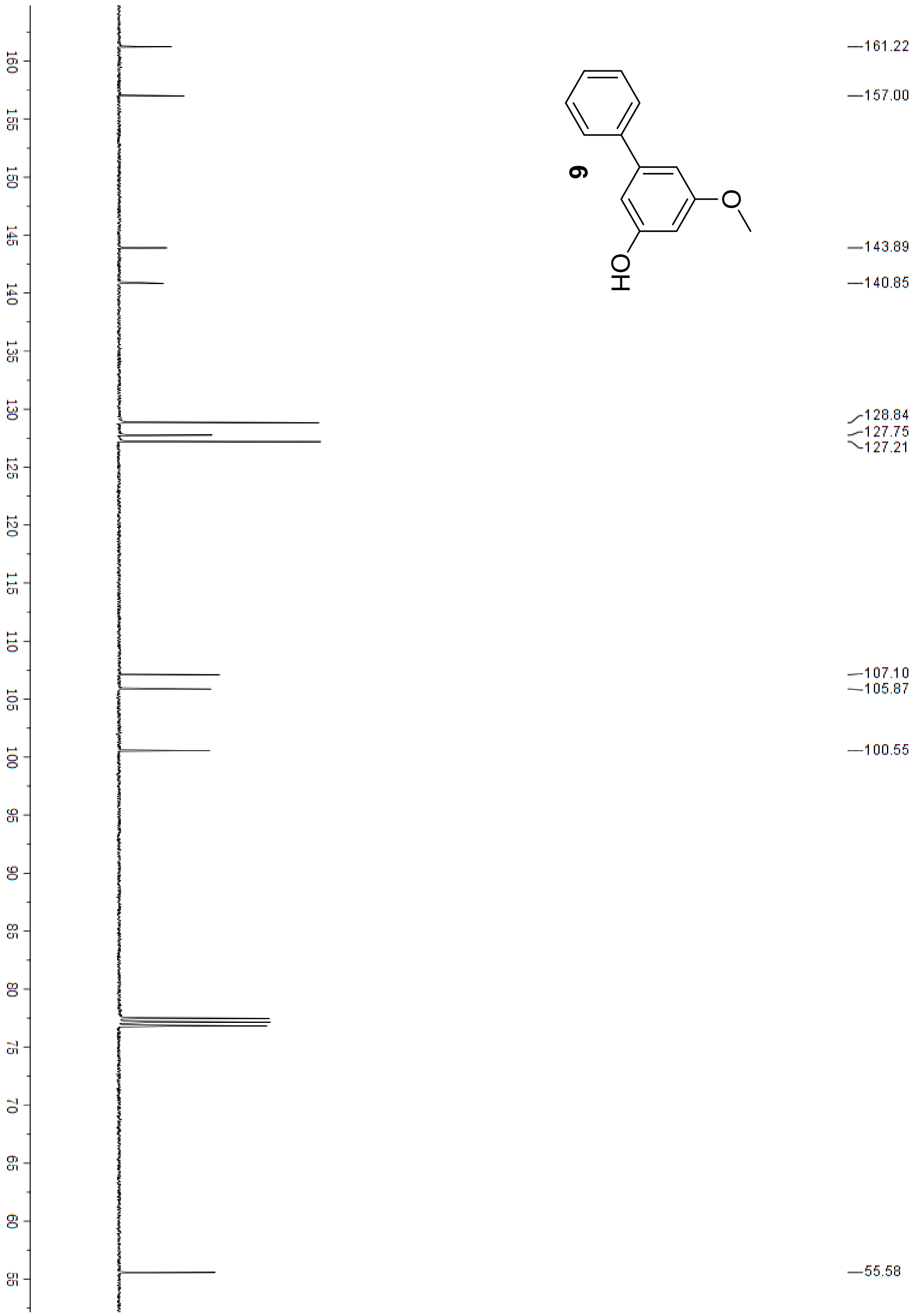

Figure S39. ^{1}H NMR spectrum of compound **6a** in $CDCl_3$

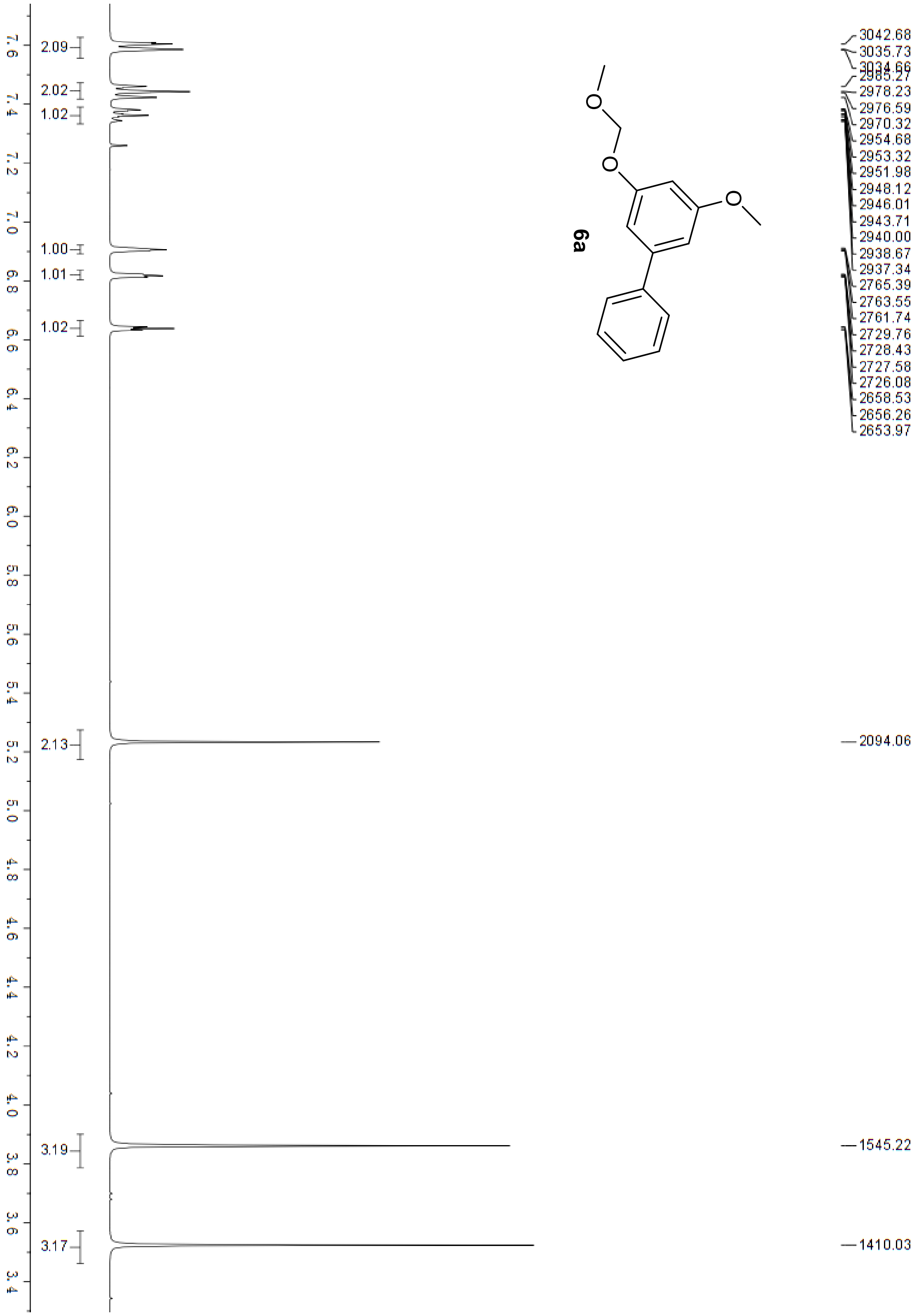

Figure S40. ^{13}C NMR spectrum of compound **6a** in $CDCl_3$

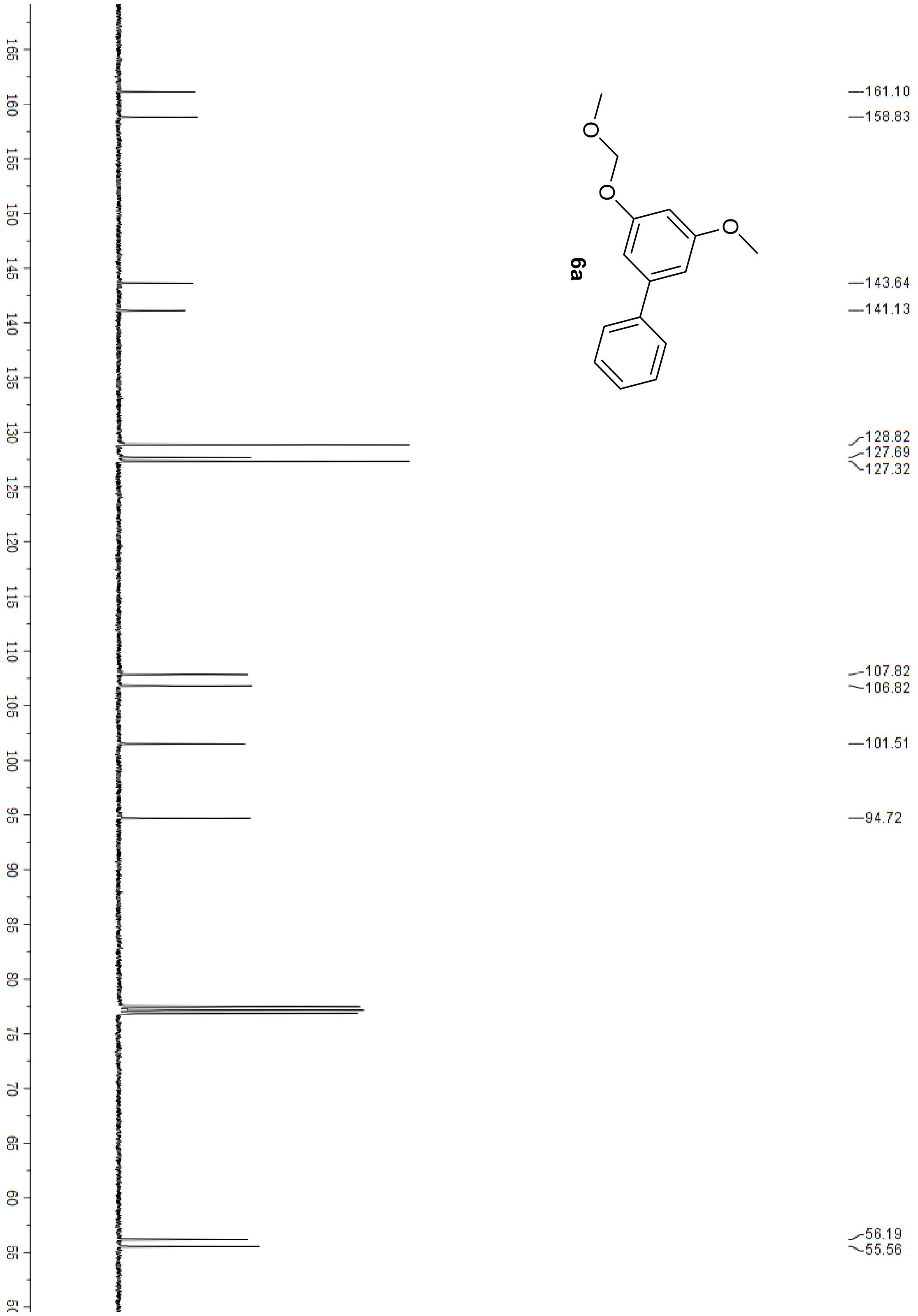

Figure S41. ^{1}H NMR spectrum of synthetic compounds **3a** + **4a** (with minor **1a** + **2a**) in $CDCl_3$

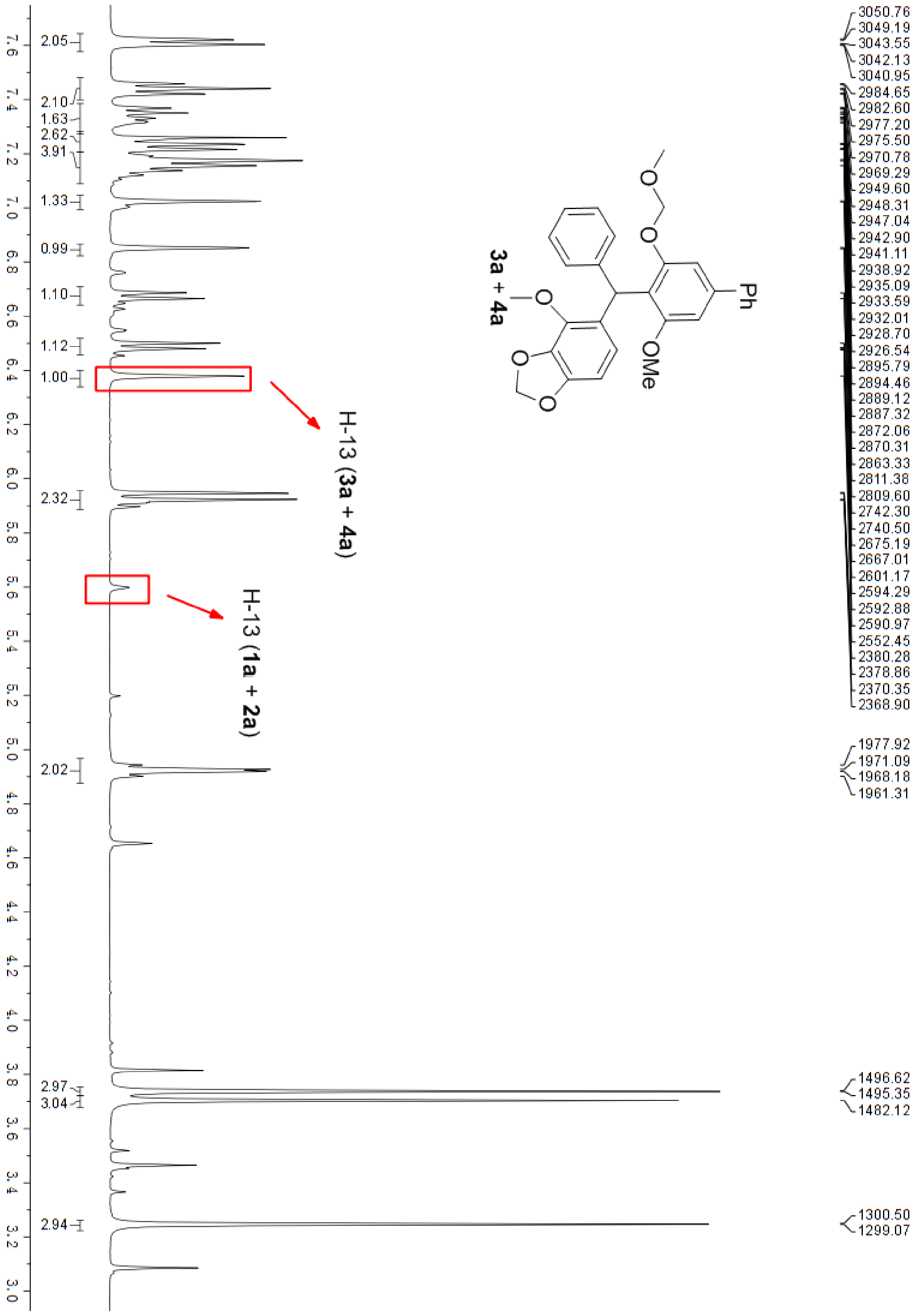

Figure S42. ${}^{13}C$ NMR spectrum of synthetic compounds **3a** + **4a** (with minor **1a** + **2a**) in $CDCl_3$

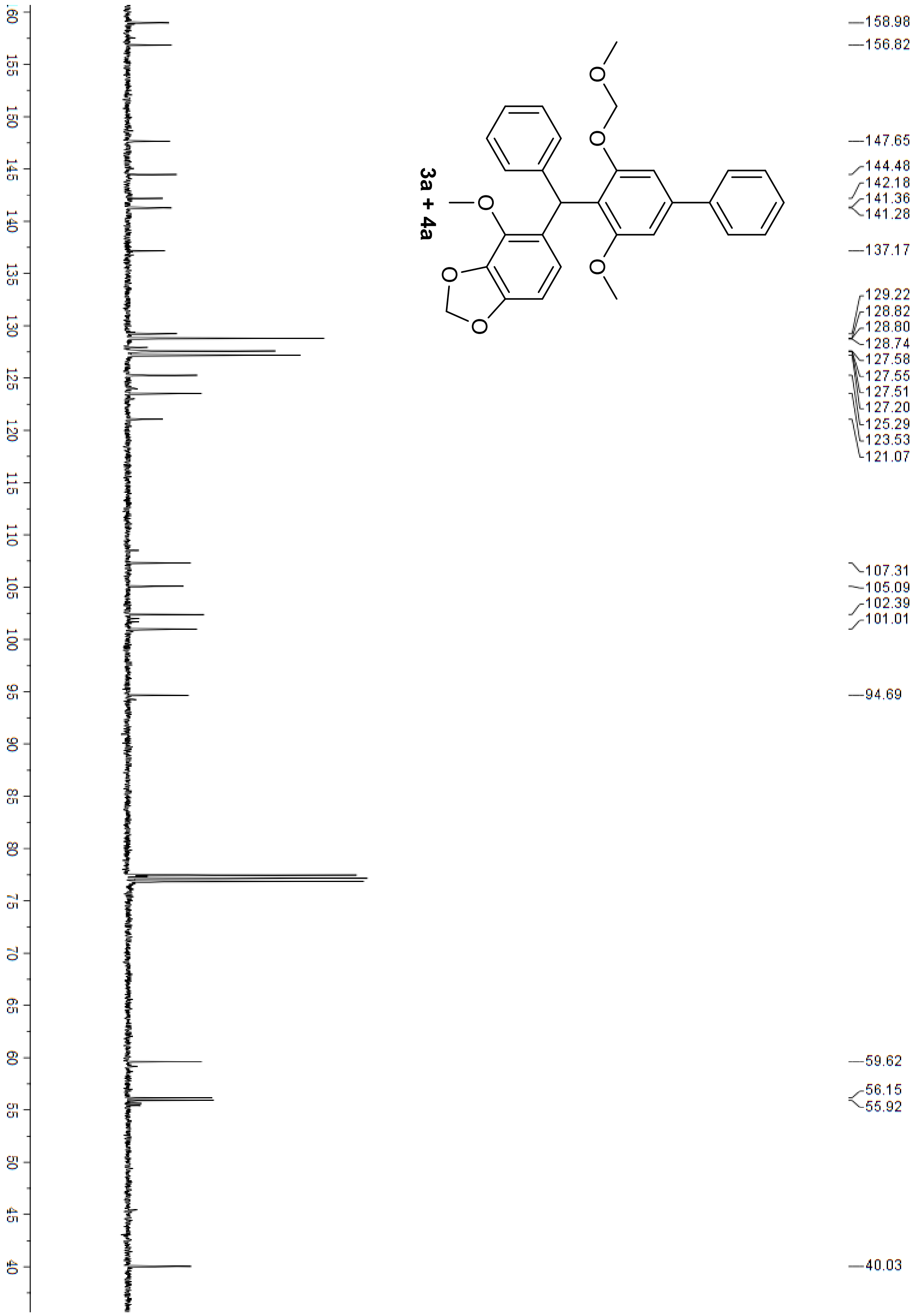

Figure S43. LC-MS of **1** (**a**), **3** (**b**) and the ethanolic crude extract (**c**)

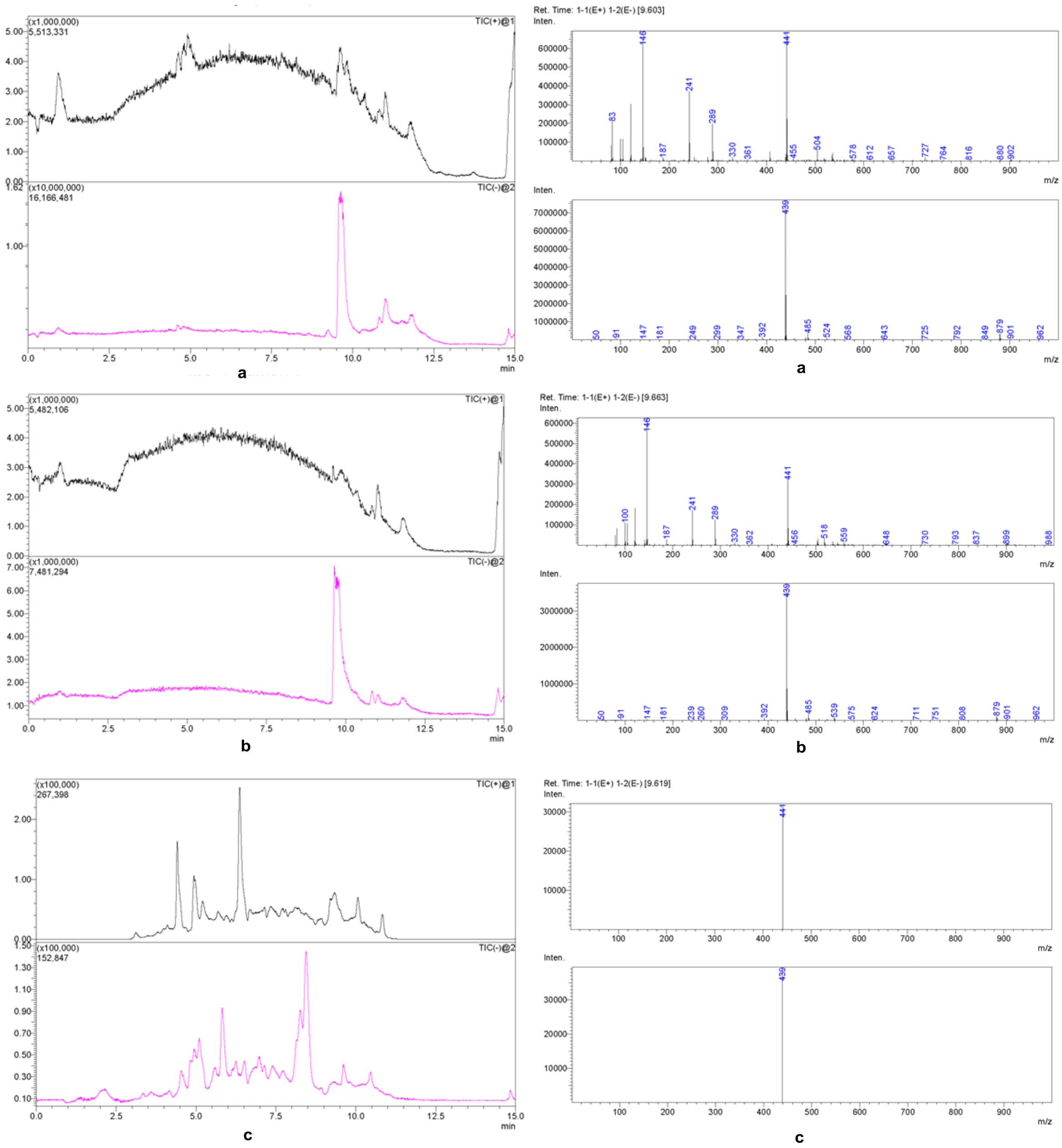

Supplement: Supplementary Materials — Table S1. Figures S1~S43: general experimental procedures, X-ray crystal data of 1 and 2, biomimetic total synthesis of 1–4, PTP1B inhibitory assay, and molecular docking of 1–4. [file 2674182.f1.pdf]
